# A modular approach to neutral P,N-ligands: synthesis and coordination chemistry

**DOI:** 10.3762/bjoc.12.83

**Published:** 2016-04-29

**Authors:** Vladislav Vasilenko, Torsten Roth, Clemens K Blasius, Sebastian N Intorp, Hubert Wadepohl, Lutz H Gade

**Affiliations:** 1 Anorganisch-Chemisches Institut, Universität Heidelberg, Im Neuenheimer Feld 270, 69120 Heidelberg, Germany

**Keywords:** P,N-ligands, phosphanylformamidines, phosphine imines, transition metal complexes

## Abstract

We report the modular synthesis of three different types of neutral κ^2^-P,N-ligands comprising an imine and a phosphine binding site. These ligands were reacted with rhodium, iridium and palladium metal precursors and the structures of the resulting complexes were elucidated by means of X-ray crystallography. We observed that subtle changes of the ligand backbone have a significant influence on the binding geometry und coordination properties of these bidentate P,N-donors.

## Introduction

P,N-ligands have been applied in a wide variety of chemical reactions ranging from hydrogenations [1–2] and allylic substitutions [3–4] to Heck reactions [5] and conjugate additions to enones [6]. Their popularity arises from the inherent electronic disparity of the phosphorus and the nitrogen donor groups, rendering one binding site a soft π-acceptor featuring a pronounced *trans* effect and the other site a hard σ-donor [7]. In addition, the steric and electronic properties of both donor groups can in principle be varied separately, rendering modular construction approaches particularly appealing [8].

**Figure 1:**
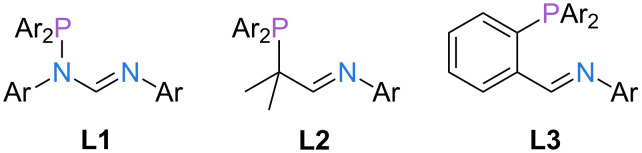
P,N-ligand frameworks studied in this work.

Driven by our recent efforts to provide easily accessible, modular ligand families [9–10], we have explored three different possibilities to expand the portfolio of P,N-ligands (**L1**–**L3**, Figure 1). We reasoned that suitable candidates should be accessible on a multigram scale in excellent yields starting from commercially available reagents and ideally involving a maximum of two steps. In addition, the resulting ligand families should provide nitrogen and phosphorus donors with varying donor strength (amidine vs imine, phosphine vs heteroatom-bound phosphorus) and different bite angles (five- vs six-membered chelate rings).

## Results and Discussion

### Ligand synthesis

**Scheme 1:**
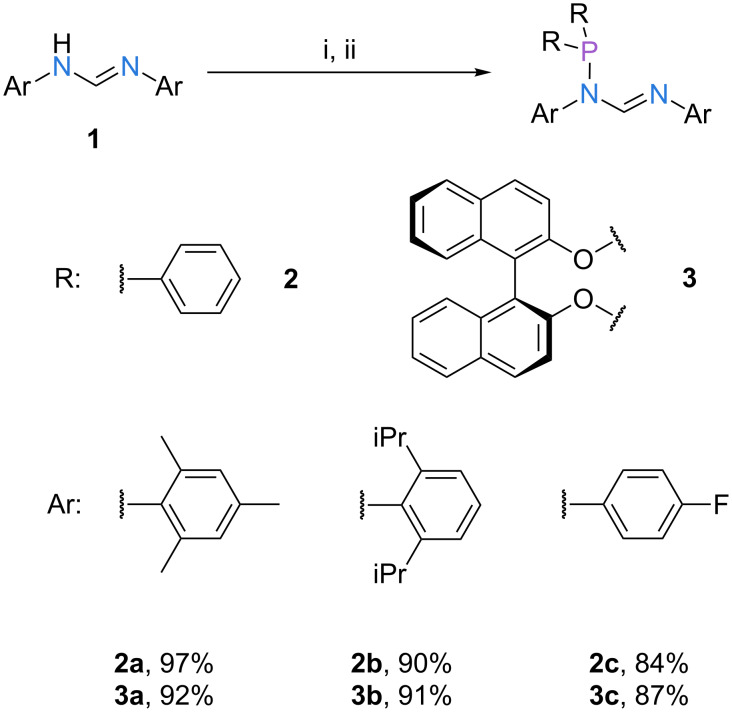
Synthesis of *N*-phosphanylformamidines **2** and **3**. Reaction conditions: (i) *t-*BuLi, THF, −78 °C to rt, 1 h; (ii) R_2_PCl, THF, −78 °C to rt, overnight.

We based the preparation of the ligand systems **L1**–**L3** on a simple construction principle, i.e., only reactions of carbon- or nitrogen-nucleophiles with chlorophosphines, and condensations of amines with aldehydes were employed. For the synthesis of the *N*-phosphanylformamidine derivatives **2** and **3** we prepared a set of three aromatic formamidines (**1a**–**c**) starting from triethyl orthoformate and aromatic amines. Low-temperature lithiation of **1** and addition to achiral (R = Ph) and axially chiral (R_2_ = BINOL) chlorophosphines R_2_PCl gave ligands **2** and **3** in excellent yields (Scheme 1) [11]. If the correct stoichiometry is maintained throughout the reaction, the resulting mixtures do not require a purification step beyond a filtration from toluene or hexane to remove residual lithium chloride. As has been pointed out by Dyer et al*.* for the structurally related *N*-phosphanylamidines, there are several distinct conformers of ligands **2a**–**c** and **3a**–**c** that can exist in solution, depending on the orientation of the phosphorus lone pair, the geometry of the C=N double bond and the orientation of the substituents of the C–N single bond [12]. Notably, all synthesized derivatives feature a single signal in the ^31^P NMR spectrum at room temperature, indicating that a relatively fast interconversion of the different conformers occurs at ambient conditions. However, with increasing steric bulk of the aromatic nitrogen substituents, a substantial line broadening is observed, indicating the rise of the isomerization barrier through steric repulsion of the neighboring groups [11].

**Scheme 2:**
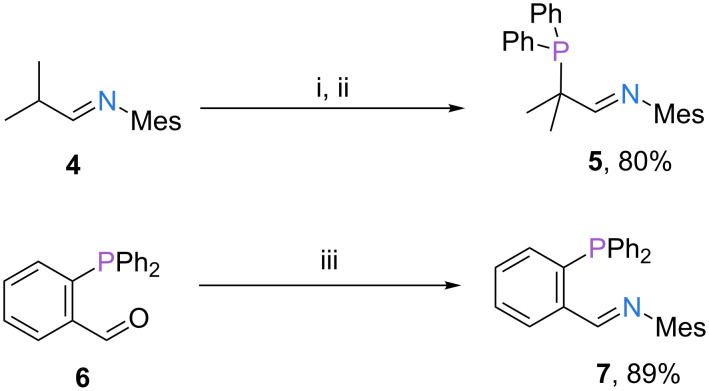
Synthesis of phosphanylformamidines **5** and **7**. Reaction conditions: (i) *t-*BuLi, THF, −78 °C to rt, 1 h; (ii) Ph_2_PCl, THF, −78 °C to rt, overnight; (iii) NH_2_Mes, toluene, 135 °C, 20 h.

The methodology for the synthesis of compounds **2** and **3** was also applied to the preparation of phosphine imine ligand **5**. Instead of a formamidine precursor, imine **4** was employed as the nucleophile. Deprotonation of **4** at low temperature and addition of the resulting C-nucleophile to chlorodiphenylphosphine gave the desired product in good yield (Scheme 2) [13]. This modification renders the phosphorus donor site more electron rich and also results in a more robust P–C bond compared to ligands **2** and **3**.

For the preparation of ligand **7** the order of bond formations of the previous protocols was reversed, that means, the carbon–nitrogen bond was formed in the second step of the synthesis. Condensation of aldehyde **6** with mesitylamine gave ligand **7** in good yield [14–16].

The connectivity of the ligands **2**, **3**, **5** and **7** imposes a varying level of rigidity on these bidentate donors, with distinct consequences for their coordination chemistry (vide infra).

### Complex synthesis

**Scheme 3:**
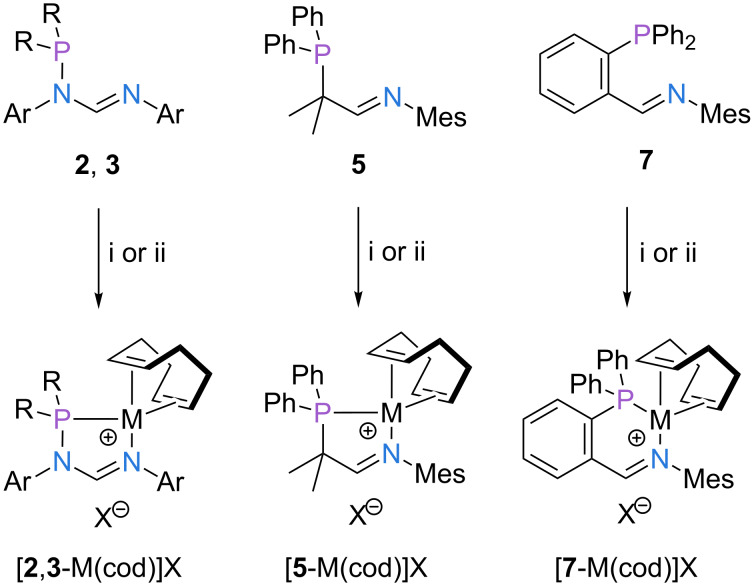
Synthesis of complexes [**2**-M(cod)]X, [**3**-M(cod)]X, [**5**-M(cod)]X and [**7**-M(cod)]X. M = Rh, Ir; X = BF_4_^−^ or OTf^−^. Reaction conditions: (i) [Rh(cod)_2_]BF_4_, DCM, rt, 30 min; (ii) [Ir(cod)Cl]_2_, AgBF_4_ or AgOTf, DCM, rt, 30 min. Ar = mesityl, 2,6-diisopropylphenyl, 4-fluorophenyl; R = phenyl or BINOL. Yields: [**2a**-Rh(cod)]BF_4_ = 85%, [**2b**-Rh(cod)]BF_4_ = 75%, [**2c**-Rh(cod)]BF_4_ = 88%, [**3a**-Rh(cod)]BF_4_ = 74%, [**3b**-Rh(cod)]BF_4_ = 90%, [**3c**-Rh(cod)]BF_4_ = 81%, [**2a**-Ir(cod)]BF_4_ = 77%, [**2b**-Ir(cod)]BF_4_ = 80%, [**2c**-Ir(cod)]BF_4_ = 88%, [**3a**-Ir(cod)]BF_4_ = 65%, [**3b**-Ir(cod)]BF_4_ = 81%, [**3c**-Ir(cod)]BF_4_ = 82%, [**5**-Rh(cod)]BF_4_ = 52%, [**5**-Ir(cod)]OTf = 67%, [**7**-Rh(cod)]BF_4_ = 92%, [**7**-Ir(cod)]BF_4_ = 85%.

In the next step of our study we set out to explore the coordination chemistry and structural properties of the synthesized ligands with rhodium(I/III), iridium(I/III) and palladium(II) precursors. Reaction of ligands **2** and **3** with (i) preformed [Rh(cod)_2_]BF_4_ and (ii) stoichiometric amounts of [Ir(cod)Cl]_2_ in the presence of AgBF_4_ gave the corresponding rhodium(I) and iridium(I) complexes [**2**,**3**-M(cod)]BF_4_ in good to excellent yields (Scheme 3). In a similar fashion, analogous complexes of ligands **5** and **7** were prepared.

**Figure 2:**
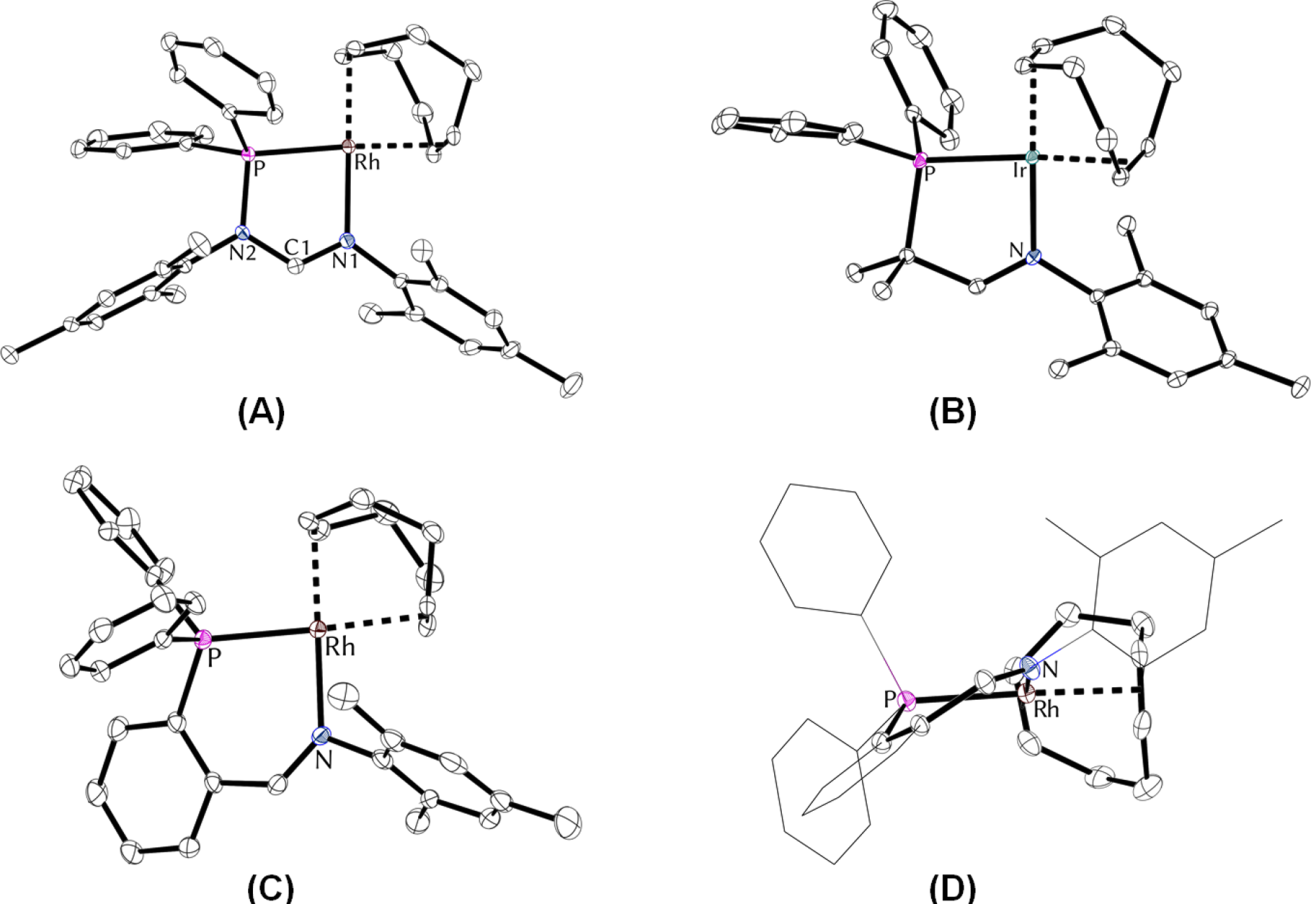
Molecular structures of [**2a**-Rh(cod)]^+^ (A), [**5**-Ir(cod)]^+^ (B), and [**7**-Rh(cod)]^+^ (C,D). Anisotropic displacement ellipsoids are set at the 50% probability level. Hydrogen atoms have been omitted for clarity. For selected bond lengths and angles see Table 1.

It should be noted that ligands **2**, **5**, and **7** feature a distinct shift of the ^31^P NMR resonance to lower fields upon complexation of a rhodium(I) or iridium(I) center, whereas a shift to higher fields is observed for ligand **3** [11]. We were able to obtain single crystals of compounds [**2a**-Rh(cod)]BF_4_, [**2b**-Rh(cod)]BF_4_, of the corresponding iridium complexes and of complexes [**5**-Rh(cod)]BF_4_, [**5**-Ir(cod)]OTf and [**7**-Rh(cod)]BF_4_ suitable for X-ray analysis by layering solutions of the complexes in dichloromethane with toluene and pentane. Instructive examples of the structural properties of selected complexes are illustrated in Figure 2. An overview of characteristic bonding properties of all crystallized compounds can be found in Table 1.

**Table 1:** Selected structural parameters of the crystallized complexes^a^.

	P–M [Å]	N–M [Å]	α^b^ [°]	β^c^ [°]

[**2a**-Rh(cod)]BF_4_	2.2338(10) [2.2351(10)]	2.0890(19) [2.0885(19)]	122.25(19) [122.35(19)]	81.69(5) [81.60(5)]
[**2b**-Rh(cod)]BF_4_	2.2487(10) [2.2452(10)]	2.0981(18) [2.0907(18)]	122.57(17) [122.55(17)]	81.42(5) [81.75(5)]
[**2a**-Ir(cod)]BF_4_	2.2447(18) [2.2389(17)]	2.083(6) [2.082(6)]	122.4(7) [122.3(6)]	81.49(16) [81.94(16)]
[**2b**-Ir(cod)]BF_4_	2.2522(7) [2.2473(7)]	2.088(2) [2.080(2)]	123.1(3) [122.9(3)]	81.83(7) [81.89(7)]
[**5**-Rh(cod)]BF_4_	2.2580(10)	2.1128(12)	122.24(11)	81.49(3)
[**5**-Ir(cod)]OTf	2.2698(9)	2.1087(15)	122.33(13)	81.59(4)
[**7**-Rh(cod)]BF_4_	2.2653(12) [2.2587(12), 2.2654(12)]	2.131(4) [2.129(4), 2.155(4)]	129.1(4) [128.9(4), 130.0(4)]	86.65(10) [87.32(10), 87.66(10)]

^a^Where applicable, values for crystallographically independent molecules are provided in square brackets. ^b^α Denotes the N–C–N angle (complexes derived from ligands **2**) or the N–C–C angle (complexes derived from ligands **5** and **7**), respectively. ^c^β Denotes the N–M–P angle.

The five-membered metallacyclic derivatives of ligands **2** and **5** feature an almost planar κ^2^-P,N chelate ring with bite angles of approx. 81–82°. Notably, increasing the number of atoms that are part of the chelate ring leads to a slightly folded structure with only minor changes in the bite angle (87°). The nitrogen-attached aryl substituent typically displays an orientation which is perpendicular to the plane of the metallacycle [17]. The P–M bond lengths in all complexes are within the expected range of 2.23–2.27 Å, as are the metal−imine bonds (2.08–2.15 Å) [18–19]. The close structural resemblance between the rhodium and iridium complexes is notable and can be attributed to the similar atomic radii of these metals [20].

Moreover, the N–C–N angles of the formamidine unit closely resemble those found in related amidinium salts, thus indicating that no significant ring strain is present. The M−C_cod_ bonds located *trans* to the P atoms are slightly longer (av 2.11 Å) than the M−C_cod_ bonds *trans* to the imino group (av 2.04 Å), suggesting a dominant *trans* influence of the phosphorus donor function compared to the imino group. The differing bond lengths within the N−C−N unit (mean 1.30 vs 1.35 Å) may indicate little or no delocalization of the positive charge via the metallacycle. Remarkably, introduction of the sterically more demanding 2,6-di(isopropyl)phenyl group as the *N*-aryl substituent induced only slight structural changes (complexes of ligand **2a** vs **2b**), mostly in the orientation of the phenyl rings of the phosphorus donor and the position of the cyclooctadiene co-ligand.

**Figure 3:**
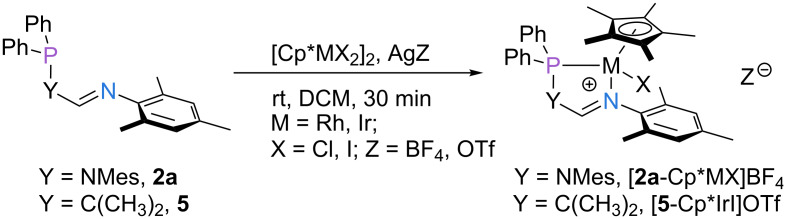
Coordination of ligands **2a** and **5** to Rh(III) and Ir(III) precursors. Yields: [**2a**-Cp*RhCl]BF_4_ = 87%, [**2a**-Cp*IrI]BF_4_ = 76%, [5-Cp*IrI]OTf = 80%.

**Figure 4:**
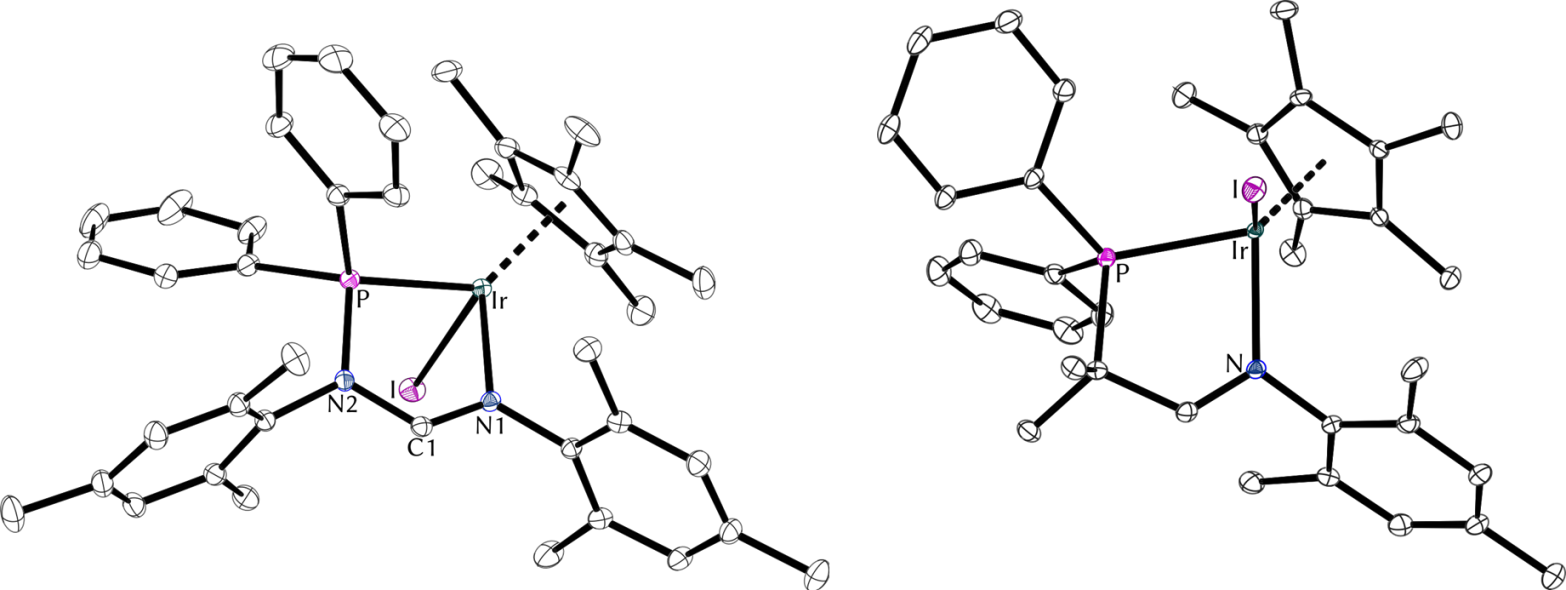
Molecular structures of [**2a**-Cp*IrI]^+^ (left) and [**5**-Cp*IrI]^+^ (right). Anisotropic displacement ellipsoids set at the 50% probability level. Hydrogen atoms have been omitted for clarity. For selected bond lengths and angles see Table 2.

To further explore the binding properties of the P,N-ligand scaffold, ligands **2a** and **5** were reacted with the Lewis-acidic precursors [Cp*RhCl_2_]_2_ and [Cp*IrI_2_]_2_ (Figure 3). As has been pointed out for the analogous Rh(I) and Ir(I) compounds, complexation is accompanied by a ^31^P NMR shift to lower fields (from 49.7 ppm to 108.9 ppm and 80.6 ppm for the Rh(III) and Ir(III) complexes of **2a**, respectively). The structures of [**2a**-Cp*IrI]BF_4_, and [**5**-Cp*IrI]BF_4_ were confirmed unambiguously by single-crystal X-ray diffraction. The solid-state structure of [**2a**-Cp*IrI]BF_4_ confirms the tetrahedral environment of the iridium center, rendering the two faces of the complex inequivalent (Figure 4, left). Similar findings were made for complex [**5**-Cp*IrI]BF_4_ (Figure 4, right). This is also reflected in the NMR spectra of both complexes, featuring a distinct set of resonances for each methyl group of the mesityl substituent.

**Table 2:** Selected structural parameters of the crystallized complexes [**2a**-Cp*IrI]BF_4_ and [**5**-Cp*IrI]OTf.

	P–Ir [Å]	N–Ir [Å]	α^a^ [°]	β^b^ [°]

[**2a**-Cp*IrI]BF_4_	2.2695(7)	2.1322(15)	122.05(14)	80.42(4)
[**5**-Cp*IrI]OTf	2.3052(8)	2.136(2)	124.4(2)	79.75(6)

^a^α Denotes the N–C–N angle ([**2a**-Cp*IrI]BF_4_) or the N–C–C angle ([**5**-Cp*IrI]OTf), respectively. ^b^β Denotes the N–Ir–P angle.

Despite the changes in the oxidation state of the metal center, only subtle deviations in the bond lengths of the chelate ligands were observed upon going from the Ir(I) to the Ir(III) complexes (in complexes of ligand **2a**: avg. 2.418 Å to 2.270 Å for the P–Ir bond and 2.083 Å to 2.132 Å for the N–Ir bond; in complexes of ligand **5**: 2.270 Å to 2.305 Å for the P–Ir bond and 2.109 Å to 2.136 Å for the N–Ir bond). Similarly, no major structural changes of the ligand backbone were detected for the different oxidation states.

**Figure 5:**
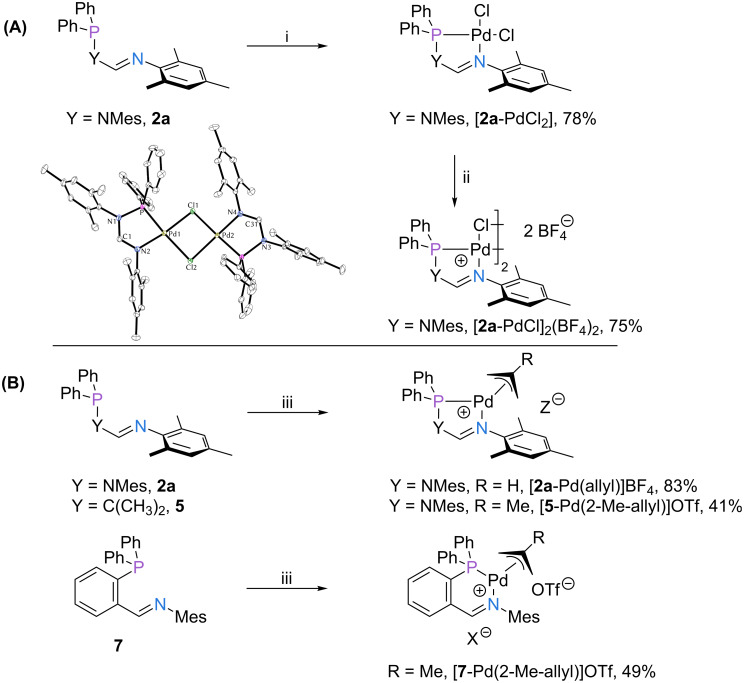
Formation of palladium complexes of ligands **2a**, **5** and **7**. (A) Formation of [**2a**-PdCl_2_] and [**2a**-PdCl]_2_(BF_4_)_2_, and molecular structure of the dimer [**2a**-PdCl]_2_^2+^. Anisotropic displacement ellipsoids set at the 50% probability level. Hydrogen atoms have been omitted for clarity. (B) Formation of Pd(allyl) complexes of ligands **2a**, **5**, and **7**. Reaction conditions: (i) DCM, [Pd(cod)Cl_2_], rt, 30 min; (ii) DCM, AgBF_4_, rt, 30 min; (iii) DCM, [Pd(allyl)Cl]_2_ or [Pd(2-Me-allyl)Cl]_2_, AgBF_4_ or AgOTf, rt, 30 min.

Furthermore, due to the importance of palladium in common transition-metal-catalyzed transformations [21], we decided to explore the reactivity of the three P,N-ligand families with the widely used precursors [Pd(cod)Cl_2_] and [Pd(allyl)Cl]_2_ (Figure 5). Addition of ligand **2a** to a solution of [Pd(cod)Cl_2_] in DCM gave complex [**2a**-PdCl_2_] in good yield. In the presence of AgBF_4_ complex [**2a**-PdCl_2_] was converted to the cationic palladium species [**2a**-PdCl]BF_4_, which is dimeric in the solid state [11]. Similarly, Pd(allyl) complexes of ligands **2a**, **5** and **7** were obtained from mixtures of the respective ligand, [Pd(allyl)Cl]_2_ and AgBF_4_ or AgOTf as the chloride scavenger in DCM (Figure 5B).

**Figure 6:**
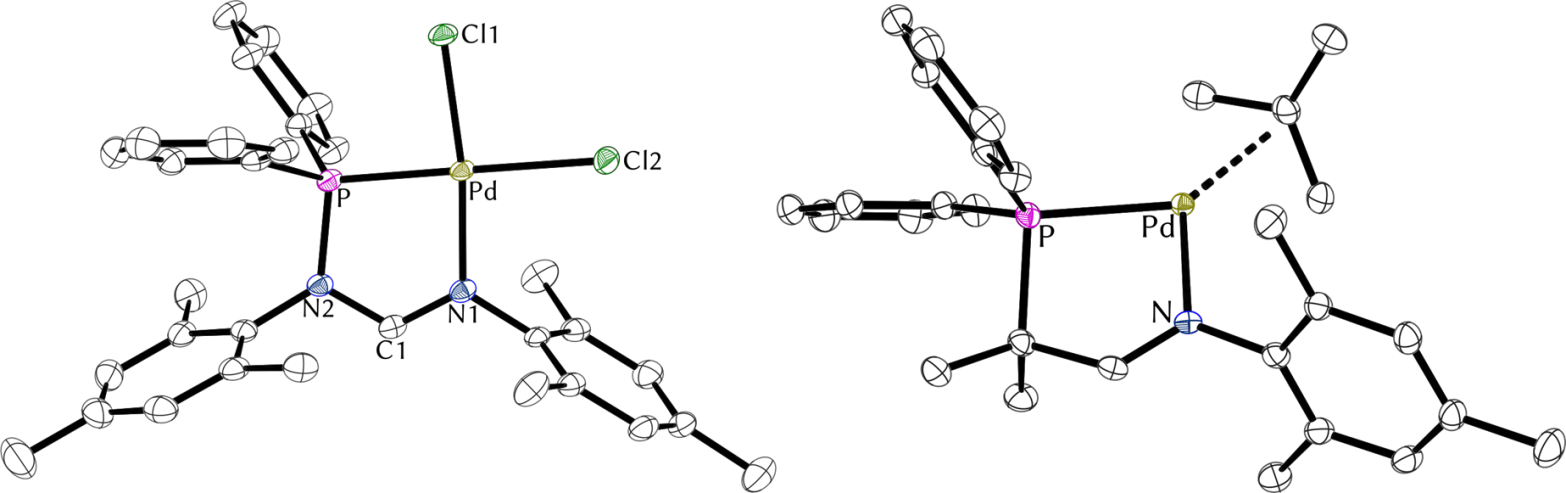
Molecular structures of [**2a**-PdCl_2_] (left) and [**5**-Pd(2-Me-allyl)]^+^ (right). Anisotropic displacement ellipsoids set at the 50% probability level. Hydrogen atoms have been omitted for clarity.

We were able to obtain single crystals of [**2a**-PdCl_2_] and [**5**-Pd(2-Me-allyl)]OTf suitable for X-ray diffraction analysis (Figure 6). For an overview of metric parameters of all palladium complexes see Table 3.

**Table 3:** Selected structural parameters of the crystallized complexes [**2a**-PdCl_2_], [**2a**-PdCl]_2_(BF_4_)_2_ and [**5**-Pd(allyl)]OTf.

	P–Pd [Å]	N–Pd [Å]	α^a^ [°]	β^b^ [°]

[**2a**-PdCl_2_]	2.1823(7)	2.043(2)	121.5(2)	83.59(7)
[**2a**-PdCl]_2_BF_4_^c^	2.1861(11) [2.1848(10)]	2.007(2) [2.019(2)]	121.4(2) [121.4(3)]	83.62(7) [83.69(7)]
[**5**-Pd(2-Me-allyl)]OTf	2.2806(14)	2.107(3)	122.2(3)	82.24(8)

^a^α Denotes the N–C–N angle ([**2a**-PdCl_2_], [**2a**-PdCl]_2_BF_4_) or the N–C–C angle ([**5**-Pd(allyl)]OTf), respectively. ^b^β Denotes the N–Pd–P angle. ^c^Values for the second palladium center of the dimer are provided in square brackets.

The solid-state structures of [**2a**-PdCl_2_] (Figure 6, left) and its cationic congener [**2a**-PdCl]_2_(BF_4_)_2_ (Figure 5) show distinct similarities. Bond lengths and bond angles of the chelate ring agree well, underlining that halide abstraction is effectively compensated by the bridging chlorides (Table 3). Significant structural deviations are only observed for the Pd–Cl bonds, where significantly longer bonds were found for the cationic species (*trans*-nitrogen 2.31 Å vs 2.33 Å and *trans*-phosphorus 2.33 Å vs 2.42 Å). As has been observed for the corresponding rhodium and iridium complexes (vide supra), the structure of complex [**5**-Pd(allyl)]OTf is slightly folded, which is also accompanied by longer P–Pd and N–Pd bonds (cf. Table 2 and Table 3).

## Conclusion

The work described in this paper shows that three related neutral κ^2^-P,N-ligand families **L1**–**L3** are accessible via straightforward condensation protocols. All ligand classes can be synthesized starting from cheap, commercially available reagents on a multigram scale. Notably, relatively small changes in the ligand backbone, i.e., C/N-exchange or increasing the chelate ring size, have a significant impact on the ligand geometry and its coordination properties. Thus, complexes of **L1** with rhodium, iridium and palladium form planar chelate rings, structures based on **L2** are slightly folded, whereas complexes of **L3** exhibit a strong deviation from planarity. In summary, these results may be utilized for the design, preparation and structural elucidation of novel late transition metal complexes. Investigations into their potential as precatalysts for organic transformations are currently underway in our laboratory and will be reported in due course.

## Experimental

### General procedure for the preparation of ligands **2**, **3** and **5**

To a solution of the formamidine or imine (15.0 mmol, 1.0 equiv) in 150 mL of THF at −78 °C was added dropwise a solution of *tert*-butyllithium in pentane (15.0 mmol, 1.0 equiv, 1.9 M). The reaction was left at this temperature for 30 min, warmed to rt and stirred for 1 h. This mixture was added to a solution of the chlorophosphine (15.0 mmol, 1.0 equiv) in 150 mL of THF at −78 °C, stirred for 30 min at this temperature and warmed to rt overnight. The solvent was removed under reduced pressure and the residue was taken up in 300 mL of toluene. The mixture was then filtered through a plug of Celite^©^ and the solvent was evaporated in vacuo yielding the desired product which was used without further purification in the subsequent metalation steps. For compound numbering and the preparation of ligand **7**, see Supporting Information File 1.

**General procedure for the preparation of metal complexes:** A solution of the ligand (100 µmol, 1.0 equiv) in 5 mL of DCM was added to the metal precursor [M]–X (100 µmol, 1.0 equiv) and the mixture was stirred for 30 minutes. At this point, the product was either isolated by layering with toluene and pentane yielding the desired neutral product or AgBF_4_ (alternatively AgOTf) (100 µmol, 1.0 equiv) was added to produce the cationic complex. The suspension was then stirred in the dark for another 30 minutes, the solid residue was filtered off and the filtrate was layered with toluene and pentane, and stored at −40 °C. This procedure yielded a powder or in several cases single crystals suitable for X-ray diffraction. The solid was then washed with pentane and dried under high vacuum for several days to remove residual solvent. Alternatively, the complexes can be synthesized starting from a preformed cationic precursor [M]–BF_4_ (or [M]–OTf). In this case, a solution of the ligand (100 µmol, 1.0 equiv) in 5 mL DCM was added to the metal precursor [M]–BF_4_ (100 µmol, 1.0 equiv). The mixture was stirred for 30 minutes, filtered, layered with toluene and pentane and stored at −40 °C. Additional purification steps were carried out as described above. For details see Supporting Information File 1.

## Supporting Information

File 1 Experimental procedures and analytical data.

File 2 Crystal structure data.
