# Supplementary material for: A modular approach to neutral P,N-ligands: synthesis and coordination chemistry
Source: Beilstein J Org Chem. 2016 Apr 29;12:846–53. doi: 10.3762/bjoc.12.83 (PMC4901889; doi:10.3762/bjoc.12.83)
Supplement: File 1 — Experimental procedures and analytical data. [file Beilstein_J_Org_Chem-12-846-s001.pdf]

Supporting Information

for

A modular approach to neutral P,N-ligands: synthesis and coordination chemistry

Vladislav Vasilenko‡, Torsten Roth‡, Clemens K. Blasius, Sebastian N. Intorp, Hubert Wadepohl and Lutz H. Gade*

Address: Anorganisch-Chemisches Institut, Universität Heidelberg, Im Neuenheimer Feld 270, 69120 Heidelberg, Germany

Email: Lutz H. Gade - lutz.gade@uni-heidelberg.de

*Corresponding author

‡These authors contributed equally.

Dedicated to the memory of Peter Hofmann.

Experimental procedures and analytical data

Contents

1 General Information 2

2 Synthetic Procedures and Analytical Data 3

  2.1 Synthesis of Ligands **2a–c** and **3a–c** 3

  2.2 Synthesis of Ligand **5** 10

  2.3 Synthesis of Ligand **7** 11

  2.4 Synthesis of Metal Complexes 12

3 VT-NMR Studies 37

4 X-Ray Crystal Structure Determinations 38

References 43

1 General Information

All manipulations, except those indicated, were carried out under exclusion of air and moisture using standard Schlenk and glove box techniques. As inert gas, Argon 5.0, purchased from Messer Group GmbH, was used after drying over Granusic© phosphorus pentoxide granulate. Solvents were dried over activated alumina columns using a solvent purification system (M. Braun SPS 800) or according to standard literature-known methods and stored in glass ampules under an argon atmosphere [1]. Toluene was distilled from sodium, *n*-pentane from sodium/potassium alloy, tetrahydrofuran, benzene and *n*-hexane from potassium, and dichloromethane and chloroform from calcium hydride. The same procedures were used to dry the deuterated solvents. Degassed solvents were obtained by three successive freeze-pump-thaw-cycles. NMR spectra were recorded on Bruker Avance (400 MHz, 600 MHz) instruments. Chemical shifts (δ) are reported in parts per million (ppm) and are referenced to residual proton solvent signals or carbon resonances [2, 3]. H_3PO_4 (^{31}P) and CCl_3F (^{19}F) were used as external standards. The following abbreviations were used: s (singlet), d (doublet), dd (doublet of doublets), t (triplet), q (quartet), sept (septet), m (multiplet), br s (broad signal). High-resolution mass spectra were acquired on Bruker ApexQe hybrid 9.4 T FT-ICR (ESI, DART) and JEOL JMS-700 magnetic sector (FAB, EI, LIFDI) spectrometers at the mass spectrometry facility of the Institute of Organic Chemistry, of the University of Heidelberg. Elemental analyses were carried out in the Microanalysis Laboratory of the Heidelberg Chemistry Department on a vario MICRO cube (Elementar). All chemicals were obtained from commercial suppliers and were used without further purification. The formamidines **1a**–**c** were prepared according to literature procedures [4–9]. Isobutyraldehyde 2,4,6-trimethylphenylimine **4** was synthesized following a standard condensation protocol [10, 11]. 2-(Diphenylphosphino)benzaldehyde **6** was obtained commercially from Sigma Aldrich (CAS 50777-76-9). Alternatively, **6** can be synthesized starting from commercially available 2-(2-bromophenyl)-1,3-dioxolane and chlorodiphenylphosphine through a lithiation, nucleophilic substitution, and deprotection sequence [12–14].

2 Synthetic Procedures and Analytical Data

2.1 Synthesis of Ligands 2a–c and 3a–c

Compounds **2a**–**c**, **3a**–**c** were synthesized following a general procedure.

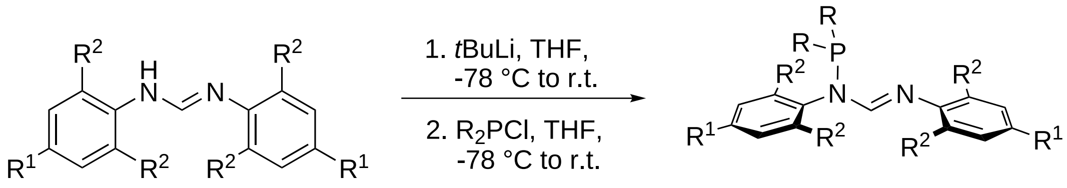

$R^1 = R^2 =$ Me, **1a**
$R^1 =$ H, $R^2 =$ *i*Pr, **1b**
$R^1 =$ F, $R^2 =$ H, **1c**

$R^1 = R^2 =$ Me, R = Ph, **2a**
$R^1 =$ H, $R^2 =$ *i*Pr, R = Ph, **2b**
$R^1 =$ F, $R^2 =$ H, R = Ph, **2c**
$R^1 = R^2 =$ Me, R = (*S*)-BINOL, **3a**
$R^1 =$ H, $R^2 =$ *i*Pr, R = (*S*)-BINOL, **3b**
$R^1 =$ F, $R^2 =$ H, R = (*S*)-BINOL, **3c**

General Procedure 1 (GP 1): To a solution of the formamidine (15.0 mmol, 1.0 equiv.) in 150 mL of THF at −78 °C was added dropwise a solution of *t*-butyl lithium in pentane (15.0 mmol, 1.0 equiv., 1.9 M). The reaction was left at this temperature for 30 min, warmed to r.t. and stirred for 1 h. This mixture was added to a solution of the chlorophosphine (15.0 mmol, 1.0 equiv.) in 150 mL of THF at −78 °C, stirred for 30 min at this temperature and warmed to r.t. over night. The solvent was removed under reduced pressure and the residue was taken up in 300 mL of toluene. The mixture was then filtered through a plug of Celite© and the solvent was evaporated *in vacuo* yielding the desired product as a colorless or yellow solid.

Preparation of Chiral Chlorophosphines: A procedure adapted from Cramer *et al.* was used [15]. A mixture of (*S*)-BINOL (10.0 mmol, 1.0 equiv.), freshly distilled PCl_3 (10 mL), and 3 drops of NMP was heated to reflux in 100 mL toluene for 10 min. The reaction mixture was concentrated *in vacuo* and the residue was distilled twice azeotropically with toluene to give the chiral chlorophosphine in quantitative yield. The product was used in GP 1 without further purification.

Compound 2a

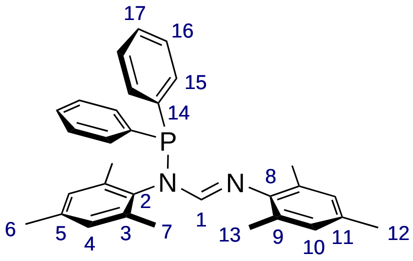

2a

yield: 4.80 g colorless solid (10.3 mmol, 97 %, GP 1).

^{1}H-NMR (399.89 MHz, THF-d_8): δ (ppm) = 1.92 (s, 6 H, H-13), 2.12 (s, 3 H, H-12), 2.16 (s, 6 H, H-7), 2.23 (s, 3 H, H-6), 6.65 (s, 2 H, H-10), 6.85 (s, 2 H, H-4), 7.34–7.45 (m, 6 H, H-16/H-17), 7.55–7.66 (m, 4 H, H-15), 7.82 (d, J = 1.8 Hz, 1 H, H-1).

^{13}C{^{1}H}-NMR (100.55 MHz, THF-d_8): δ (ppm) = 19.07 (s, 2 C, C-13), 20.05 (d, J = 1.2 Hz, 2 C, C-7), 20.57 (s, 1 C, C-12), 20.76 (d, J = 0.5 Hz, 1 C, C-6), 128.45 (s, 2 C, C-9), 128.79 (s, 2 C, C-10), 129.19 (d, J = 6.7 Hz, 4 C, C-16), 129.86 (d, J = 1.8 Hz, 2 C, C-17), 130.27 (s, 2 C, C-4), 131.37 (s, 1 C, C-11), 133.85 (d, J = 22.7 Hz, 4 C, C-15), 136.95 (d, J = 2.6 Hz, 1 C, C-5), 137.00 (d, J = 3.4 Hz, 2 C, C-3), 139.28 (d, J = 19.7 Hz, 2 C, C-14), 139.83 (d, J = 16.1 Hz, 1 C, C-2), 147.91 (d, J = 0.7 Hz, 1 C, C-8), 152.80 (d, J = 2.5 Hz, 1 C, C-1).

^{31}P{^{1}H}-NMR (161.88 MHz, THF-d_8): δ (ppm) = 49.70 (s, 1 P).

EA ($C_{31}H_{33}N_2P$): calcd. C: 80.14 %, H: 7.16 %, N: 6.03 %; found: C: 79.61 %, H: 7.33 %, N: 5.99 %.

HR-MS (DART+): $[M+H]^+ = C_{31}H_{34}N_2P^+$ calcd.: 465.2454 found: 465.2447.

Compound 2b

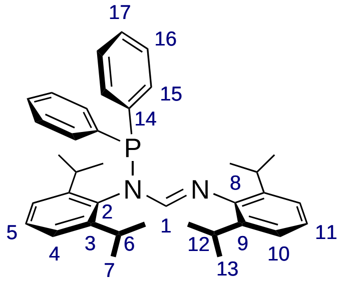

2b

yield: 6.75 g colorless solid (12.3 mmol, 90 %, GP 1).

^{1}H-NMR (600.13 MHz, THF-d_8): δ (ppm) = 0.96–1.08 (m, 18 H, H-7/H-13), 1.15–1.24 (m, 6 H, H-7/H-13), 2.94–3.06 (m, 2 H, H-12), 3.22–3.36 (m, 2 H, H-6), 6.81–6.88 (m, 1 H, H-11), 6.91–6.99 (m, 2 H, H-10), 7.15–7.21 (m, 2 H, H-4), 7.22–7.27 (m, 1 H, H-5), 7.35–7.44 (m, 6 H, H-16/H-17), 7.59–7.69 (m, 4 H, H-15), 7.82–7.91 (m, 1 H, H-1).

^{13}C{^{1}H}-NMR (150.90 MHz, THF-d_8): δ (ppm) = 24.80 (s, 2 C, C-7/C-13), 24.89 (s, 4 C, C-7/C-13), 25.55 (s, 2 C, C-7/C-13), 28.08 (s, 2 C, C-12), 29.65 (s, 2 C, C-6), 123.55 (s, 2 C, C-10), 123.88 (s, 1 C, C-11), 125.06 (d, J = 1.8 Hz, 2 C,C-4), 128.94 (d, J = 2.3 Hz, 1 C, C-5), 129.64 (d, J = 7.2 Hz, 4 C, C-16), 130.86 (s, 2 C, C-17), 134.41 (d, J = 23.7 Hz, 4 C, C-15), 138.70 (d, J = 20.1 Hz, 2 C, C-14), 139.90 (d, J = 18.6 Hz, 1 C, C-2) 140.17 (s, 2 C, C-9), 147.75 (d, J = 0.8 Hz, 1 C, C-8), 148.19 (d, J = 3.2 Hz, 2 C, C-3), 153.84 (d, J = 3.9 Hz, 1 C, C-1).

^{31}P{^{1}H}-NMR (242.94 MHz, THF-d_8): δ (ppm) = 47.51 (br s, 1 P).

EA ($C_{37}H_{45}N_2P$): calcd. C: 80.98%, H: 8.21 %, N: 5.11 %; found: C: 80.17 %, H: 8.01 %, N: 5.22 %.

HR-MS (ESI+): $[M+H]^+$= $C_{37}H_{46}N_2P^+$ calcd.: 549.3393 found: 549.3396.

Compound 2c

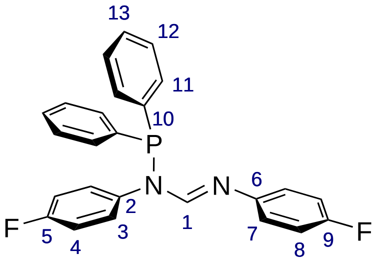

2c

yield: 7.50 g yellow oil (18.0 mmol, 84 %, GP 1).

^{1}H-NMR (600.13 MHz, THF-d_8): δ (ppm) = 6.72−6.78 (m, 2 H, H-7), 6.86−6.91 (m, 2 H, H-8), 6.96−6.99 (m, 2 H, H-4), 7.10−7.14 (m, 2 H, H-3), 7.38−7.43 (m, 6 H, H-Ar), 7.47−7.52 (m, 4 H, H-Ar), 8.07 (d, J = 3.1 Hz, 1 H, H-1).

^{13}C{^{1}H}-NMR (150.90 MHz, THF-d_8): δ (ppm) = 115.44 (d, J = 22.3 Hz, 2 C, C-8), 115.46 (dd, J = 22.6 Hz, J = 6.6 Hz, 2 C, C-4), 122.43 (d, J = 7.9 Hz, 2 C, C-7), 128.94 (d, J = 6.1 Hz, 4 C, C-Ar), 130.03 (s, 2 C, C-Ar), 130.37−130.56 (m, 2 C, C-3), 132.76−133.03 (m, 4 C, C-Ar), 136.91 (d, J = 16.6 Hz, 2 C, C-10), 139.66 (m, 1 C, C-2), 147.57 (d, J = 2.9 Hz, 1 C, C-6), 154.53 (d, J = 18.6 Hz, 1 C, C-1), 160.12 (d, J = 240.2 Hz, 1 C, C-9), 161.41 (d, J = 244.4 Hz, 1 C, C-5).

^{31}P{^{1}H}-NMR (242.94 MHz, THF-d_8): δ (ppm) = 60.64 (s, 1 P).

EA ($C_{25}H_{19}F_2N_2P$): calcd. C: 72.11 %, H: 4.60 %, N: 6.73 %; found: C: 71.49 %, H: 4.81 %, N: 6.66 %.

HR-MS (ESI+): $[M+H]^{+}$= $C_{25}H_{20}F_2N_2P^{+}$ calcd.: 417.1327 found: 417.1337.

Compound 3a

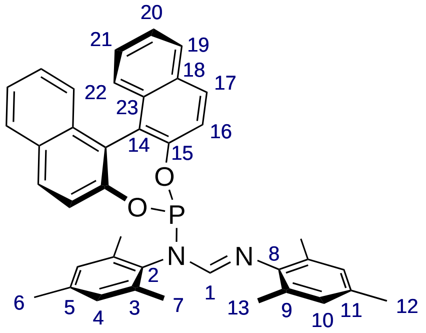

3a

yield: 9.50 g yellow solid (16.0 mmol, 92 %, GP 1).

^{1}H-NMR (600.13 MHz, THF-d_8): δ (ppm) = 1.84 (s, 6 H, H-13), 2.03 (s, 3 H, H-12), 2.29 (s, 3 H, H-7), 2.45 (s, 3 H, H-6), 2.62 (s, 3 H, H-7), 6.52 (s, 2 H, H-10), 6.99 (s, 1 H, H-4), 7.01 (s, 1 H, H-4), 7.17–7.23 (m, 2 H, H-20/H-21), 7.25–7.29 (m, 2 H, H-19/H-22), 7.35–7.39 (m, 1 H, H-20/H-21), 7.40 (d, J = 1.5 Hz, 1 H, H-1), 7.41–7.45 (m, 1 H, H-20/H-21), 7.56 (d, J = 8.6 Hz, 1 H, H-17), 7.68 (d, J = 8.8 Hz, 1 H, H-17), 7.89 (d, J = 8.4 Hz, 1 H, H-19/H-22), 7.96 (d, J = 8.6 Hz, 1 H, H-19/H-22), 8.00 (d, J = 8.6 Hz, 1 H, H-16), 8.08 (d, J = 8.8 Hz, 1 H, H-16).

^{13}C{^{1}H}-NMR (150.90 MHz, THF-d_8): δ (ppm) = 19.10 (s, 2 C, C-13), 19.31 (d, J = 3.8, 1 C, C-7), 19.81 (s, 1 C, C-7), 20.73 (s, 1 C, C-12), 21.16 (s, 1 C, C-6), 122.22 (s, 1 C, C-17), 122.31 (d, J = 1.6 Hz, 1 C, C-17), 123.65 (d, J = 2.4 Hz, 1 C, C-Ar), 124.99 (d, J = 5.5 Hz, 1 C, C-Ar), 125.40 (d, J = 5.5 Hz, 1 C, C-Ar), 125.95 (s, 1 C, C-20/C-21), 126.12 (s, 1 C, C-20/C-21), 127.31 (s, 1 C, C-20/C-21), 127.32 (s, 1 C, C-20/C-21), 127.64 (s, 1 C, C-19/C-22), 127.71 (s, 1 C, C-19/C-22), 128.12 (s, 2 C, C-9), 128.92 (s, 2 C, C-10), 128.96 (s, 1 C, C-11), 129.24 (s, 1 C, C-19/C-22), 129.43 (s, 1 C, C-19/C-22), 130.03 (m, 2 C, C-4), 131.51 (s, 1 C, C-16), 131.88 (d, J = 1.3 Hz, 1 C, C-16), 132.18 (s, 1 C, C-Ar), 133.43 (d, J = 1.0 Hz, 1 C, C-Ar), 133.69 (d, J = 1.5 Hz, 1 C, C-Ar), 134.88 (d, J = 18.6 Hz, 1 C, C-2), 138.05 (d, J = 4.3 Hz, 1 C, C-Ar), 138.51 (d, J = 2.9 Hz, 1 C, C-Ar), 138.75 (d, J = 4.4 Hz, 1 C, C-Ar), 146.80 (s, 1 C, C-8), 148.25 (br s, 1 C, C-1), 149.20 (d, J = 6.5 Hz, 1 C, C-Ar), 149.43 (s, 1 C, C-Ar).

^{31}P{^{1}H}-NMR (242.94 MHz, THF-d_8): δ (ppm) = 135.44 (s, 1 P).

EA ($C_{39}H_{45}N_2O_2P$): calcd. C: 78.77 %, H: 5.93 %, N: 4.71 %; found: C: 78.81 %, H: 6.16 %, N: 4.57 %.

HR-MS (ESI+): $[M+H]^+$= $C_{39}H_{46}N_2O_2P^+$ calcd.: 595.2509 found: 595.2488.

Compound 3b

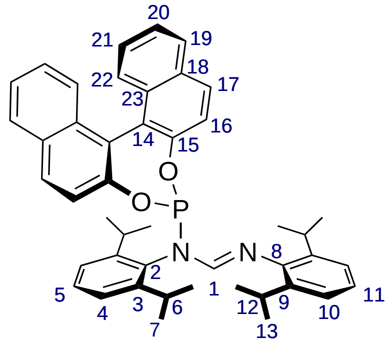

3b

yield: 10.8 g yellow solid (15.9 mmol, 91 %, GP 1).

^{1}H-NMR (600.13 MHz, THF-d_8): δ (ppm) = 0.86 (d, J = 6.6 Hz, 6 H, H-13), 1.07 (d, J = 6.9 Hz, 6 H, H-13), 1.27 (d, J = 6.8 Hz, 6 H, H-7), 1.40 (d, J = 6.4 Hz, 3 H, H-7), 1.43 (d, J = 6.7 Hz, 3 H, H-7) 2.89–3.03 (m, 2 H, H-12), 3.48 (sept, J = 6.8 Hz, 1 H, H-6), 3.62 (sept, J = 6.8 Hz, 1 H, H-6), 6.72–6.78 (m, 1 H, H-Ar), 6.80–6.86 (m, 2 H, H-Ar), 7.16–7.31 (m, 6 H, H-Ar), 7.30–7.34 (m, 2 H, H-Ar), 7.34–7.38 (m, 1 H, H-Ar), 7.40–7.46 (m, 1 H, H-17), 7.42 (d, J = 1.1 Hz, 1 H, H-1), 7.58 (d, J = 8.8 Hz, 1 H, H-17), 7.80 (d, J = 8.2 Hz, 1 H, H-20/H-21), 7.84 (d, J = 8.8 Hz, 1 H, H-19/H-22), 7.98 (d, J = 8.3 Hz, 1 H, H-16), 8.09 (d, J = 8.8 Hz, 1 H, H-16).

^{13}C{^{1}H}-NMR (150.90 MHz, THF-d_8): δ (ppm) = 23.96 (br s, 1 C, C-7), 24.43 (s, 2 C, C-13), 24.53 (s, 2 C, C-13), 24.72 (br s, 1 C, C-7), 25.59 (br s, 1 C, C-7), 26.14 (br s, 1 C, C-7), 28.29 (s, 2 C, C-12), 29.76 (s, 1 C, C-6), 29.81 (s, 1 C, C-6), 121.96 (s, 1 C, C-17), 122.28 (s, 1 C, C-17), 123.26 (s, 2 C, C-Ar), 123.44 (d, J = 1.8 Hz, 1 C, C-Ar), 124.08 (s, 1 C, C-Ar), 124.81 (s, 1 C, C-Ar), 125.16 (d, J = 1.5 Hz, 1 C, C-Ar), 125.23 (d, J = 5.5 Hz, 1 C, C-Ar), 126.05 (s, 1 C, C-Ar), 126.33 (s, 1 C, C-Ar) 127.41 (s, 1 C, C-Ar), 127.48 (s, 1 C, C-Ar), 127.67 (s, 1 C, C-Ar), 127.78 (s, 1 C, C-Ar), 129.26 (s, 1 C, C-20/C-21), 129.55 (s, 1 C, C-16), 129.97 (d, J = 1.9 Hz, 1 C, C-Ar), 132.12 (s, 1 C, C-Ar), 131.72 (br s, 1 C, C-19/C-22), 132.09 (s, 1 C, C-16), 132.12 (s, 1 C, C-Ar), 133.09 (s, 1 C, C-Ar), 133.70 (d, J = 1.0 Hz, 1 C, C-Ar), 133.85 (d, J = 1.5 Hz, 1 C, C-Ar), 134.60 (d, J = 16.0 Hz, 1 C, C-Ar) 139.64 (s, 2 C, C-Ar), 146.91 (s, 1 C, C-Ar), 149.17–149.34 (m, 2 C, C-Ar), 149.46 (s, 1 C, C-Ar), 149.74 (d, J = 6.7 Hz, 1 C, C-Ar) 150.39 (br s, 1 C, C-1).

^{31}P{^{1}H}-NMR (242.94 MHz, THF-d_8): δ (ppm) = 136.34 (s, 1 P).

EA ($C_{45}H_{47}N_2O_2P$): calcd. C: 79.62 %, H: 6.98 %, N: 4.13 %; found: C: 78.98 %, H: 6.91 %, N: 4.10 %.

HR-MS (ESI+): $[M+H]^+$= $C_{45}H_{48}N_2O_2P^+$ calcd.: 679.3448 found: 679.3454.

Compound 3c

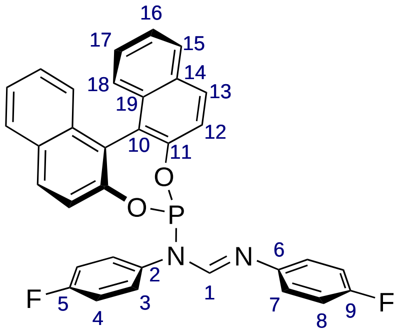

3c

yield: 8.32 g colorless solid (15.2 mmol, 87 %, GP 1).

^{1}H-NMR (600.13 MHz, CD_2Cl_2): δ (ppm) = 6.14–6.26 (m, 2 H, H-7), 6.54–6.68 (m, 2 H, H-8), 7.07–7.15 (m, 2 H, H-4) 7.28–7.41 (m, 6 H, H-Ar), 7.44 (d, J = 8.4 Hz, 1 H, H-13), 7.46–7.50 (m, 1 H, H-16/H-17), 7.52–7.56 (m, 1 H, H-16/H-17), 7.58 (d, J = 8.8 Hz, 1 H, H-13), 7.68 (d, J = 1.8 Hz, 1 H, H-1), 7.95–8.02 (m, 2 H, H-Ar), 8.02 (d, J = 8.4 Hz, 1 H, H-12), 8.06 (d, J = 8.8 Hz, 1 H, H-12).

^{13}C{^{1}H}-NMR (150.90 MHz, CD_2Cl_2): δ (ppm) = 115.26 (d, J = 22.3 Hz, 2 C, C-8), 116.33 (d, J = 22.6 Hz, 2 C, C-4), 120.81 (d, J = 8.8 Hz, 1 C, C-Ar), 121.31 (d, J = 1.5 Hz, 1 C, C-Ar), 121.63 (s, 1 C, C-Ar), 122.16 (d, J = 8.1 Hz, 2 C, C-7), 123.75 (d, J = 2.1 Hz, 1 C, C-Ar), 123.84 (d, J = 5.1 Hz, 1 C, C-Ar), 125.60 (s, 1 C, C-Ar), 125.73 (s, 1 C, C-Ar), 126.82 (s, 1 C, C-Ar), 126.93 (s, 1 C, C-Ar), 126.99 (s, 2 C, C-Ar), 128.40 (s, 1 C, C-Ar), 128.63 (s, 1 C, C-Ar), 128.65 (s, 1 C, C-Ar), 130.58 (dd, J = 6.0 Hz, J = 8.8 Hz, 2 C, C-3), 130.78 (s, 1 C, C-12), 131.16 (s, 1 C, C-12), 132.78 (d, J = 1.2 Hz, 1 C, C-Ar), 132.85 (d, J = 1.2 Hz, 1 C, C-Ar), 133.90 (dd, J = 16.3 Hz, J = 2.9 Hz, 1 C, C-2), 146.32 (dd, J = 1.1 Hz, J = 2.6 Hz, 1 C, C-6), 147.50 (d, J = 4.2 Hz, 1 C, C-Ar), 148.08 (d, J = 1.8 Hz, 1 C, C-Ar), 149.34 (d, J = 6.6 Hz, 1 C, C-1), 159.94 (d, J = 241.9 Hz, 1 C, C-9), 161.90 (d, J = 246.9 Hz, 1 C, C-5).

^{31}P{^{1}H}-NMR (242.94 MHz, CD_2Cl_2): δ (ppm) = 140.44 (s, 1 P).

EA ($C_{33}H_{21}F_2N_2O_2P$): calcd. C: 72.57 %, H: 3.87 %, N: 5.13 %; found: C: 71.99 %, H: 3.98 %, N: 5.34 %.

HR-MS (ESI+): $[M+H]^+ = C_{33}H_{22}F_2N_2O_2P^+$ calcd.: 547.1382 found: 547.1384.

2.2 Synthesis of Ligand 5

This compound was synthesized according to an adapted literature procedure [11]:

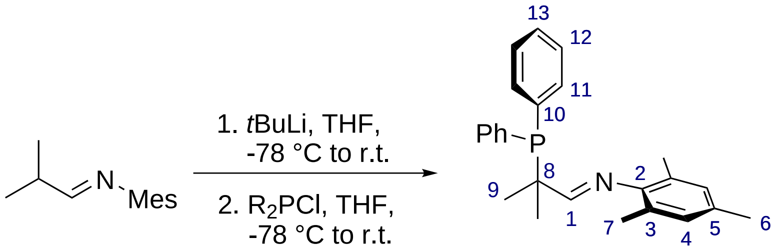

5

Isobutyraldehyde 2,4,6-trimethylphenylimine (2.01 g, 10.6 mmol, 1.0 equiv.) was dissolved in 50 mL of THF and cooled to −78 °C. A solution of *t*-butyl lithium (1.7 M in hexanes, 6.24 mL, 10.6 mmol, 1.0 equiv.) was added dropwise, the mixture was allowed to warm to r.t., and stirred for 1 h at this temperature. A solution of chlorodiphenylphosphine (2.34 g, 1.90 mL, 10.6 mmol, 1.0 equiv.) in 50 mL of THF was cooled to −78 °C and the lithiated imine was added dropwise. The mixture was stirred over night and the volatiles were removed under reduced pressure. The residue was dried thoroughly, extracted with toluene and filtered through a plug of Celite©. The clear yellow filtrate was concentrated under reduced pressure, yielding an orange-brown oil (3.17 g, 8.48 mmol, 80 %).

^{1}H-NMR (600.13 MHz, CDCl$_3$): δ (ppm) = 1.46 (d, J = 13.4 Hz, 6 H, H-9), 1.96 (s, 6 H, H-7), 2.21 (s, 3 H, H-6), 6.78 (s, 2 H, H-4), 7.30-7.31 (m, 6 H, H-Ar), 7.56-7.57 (m, 4 H, H-Ar), 7.61 (d, J = 2.1 Hz, 1 H, H-1).

^{13}C-NMR (150.90 MHz, CDCl$_3$): δ (ppm) = 18.70 (s, 2 C, C-7), 20.79 (s, 1 C, C-6), 24.32 (d, J = 16.7 Hz, 2 C, C-9), 40.89 (d, J = 18.1 Hz, 1 C, C-8), 127.22 (s, 2 C, C-3), 128.36 (d, J = 6.8 Hz, 4 C, C-12), 128.88 (s, 2 C, C-4), 129.06 (s, 2 C, C-13), 132.85 (s, 1 C, C-5), 134.72 (d, J = 19.4 Hz, 4 C, C-11), 135.23 (d, J = 17.6 Hz, 2 C, 10), 148.33 (s, 1 C, C-2), 171.94 (d, J = 5.0 Hz, 1 C, C-1).

^{31}P{^{1}H}-NMR (242.92 MHz, CDCl$_3$): δ (ppm) = 17.41 (s, 1 P).

HR-MS (ESI+): $[M]^+ = C_{25}H_{28}NP^+$ calcd.: 373.1954 found: 373.1932.

2.3 Synthesis of Ligand 7

This compound was synthesized according to a modified literature procedure [16–18]:

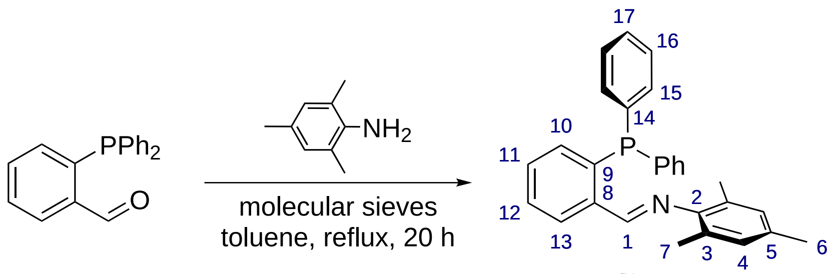

2-(Diphenylphosphino)benzaldehyde (2.09 g, 7.20 mmol, 1.0 equiv.) and mesitylamine (1.01 g, 7.44 mmol, 1.03 mmol) were dissolved in 40 mL toluene. The orange solution was heated to 135 °C for 20 h under a dropping funnel filled with molecular sieves. Evaporating the solvent *in vacuo* yielded the product as a yellow solid, which was used in following syntheses without further purification (2.60 g, 6.38 mmol, 89 %).

^{1}H-NMR (600.13 MHz, CDCl$_3$): δ (ppm) = 1.84 (s, 6 H, H-7), 2.25 (s, 3 H, H-6), 6.81 (s, 2 H, H-4), 6.91–6.95 (m, 1 H, H-Ar), 7.19 (d, J = 7.0 Hz, 1 H, H-Ar), 7.23–7.31 (m, 5 H, H-Ar), 7.31–7.37 (m, 5 H, H-Ar), 7.39 (t, J = 7.7 Hz, 1 H,H-Ar), 7.50 (t, J = 7.6 Hz, 1 H, H-Ar), 8.90 (d, J = 5.6 Hz, 1 H, H-Ar).

^{13}C-NMR (150.90 MHz, CDCl$_3$): δ (ppm) = 18.02 (s, 2 C, C-7), 20.86 (s, 1 C, C-6), 127.22 (s, 2 C, C-3), 127.71 (d, J = 4.7 Hz, 1 C, C-10), 128.63 (s, 2 C, C-4), 128.79 (d, J = 7.1 Hz, 4 C, C-16), 129.07 (s, 2 C, C-17), 129.11 (s, 1 C, C-11), 131.06 (s, 1 C, C-12/C-13), 132.97 (s, 1 C, C-5), 133.47 (s, 1 C, C-12/C-13), 134.24 (d, J = 20.1 Hz, 4 C, C-15), 136.38 (d, J = 10.0 Hz, 2 C, C-14), 138.63 (d, J = 19.9 Hz, 1 C, C-9), 139.47 (d, J = 17.5 Hz, 1 C, C-8), 148.61 (s, 1 C, C-2), 161.40 (d,J = 23.6 Hz, 1 C, C-1).

^{31}P-NMR (242.94 MHz, CDCl$_3$): δ (ppm) = −14.63 (s, 1 P).

HR-MS (ESI+): $[M]^+ = C_{28}H_{26}NP^+$ calcd.: 407.1797 found: 407.1812.

2.4 Synthesis of Metal Complexes

All metal complexes were synthesized following General Procedures 3 and 4 (GP 2 top, GP 3 bottom). In GP 2 and GP 3 the following metal precursors were used: [Pd(cod)Cl_2], [Pd(allyl)Cl]$_2$, [Cp*RhCl_2]$_2$, [Cp*IrI_2]$_2$, [Rh(cod)$_2$]BF_4, [Ir(cod)Cl]$_2$.

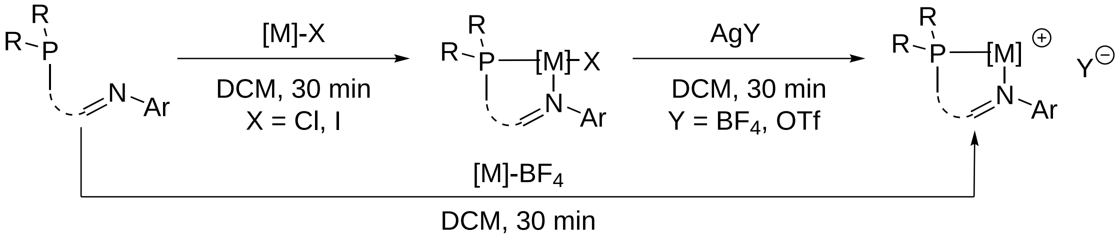

General Procedure 2 (GP 2): A solution of the ligand (100 µmol, 1.0 equiv.) in 5 mL of DCM was added to the metal precursor [M]−X (100 µmol, 1.0 equiv.) and the mixture was stirred for 30 minutes. At this point, the product was either isolated by layering with toluene and pentane yielding the desired neutral product or $AgBF_4$ (100 µmol, 1.0 equiv.) was added to produce the cationic derivative. The suspension was then stirred in the dark for another 30 minutes, the solid residue was filtered off and the filtrate was layered with toluene and pentane, and stored at −40 °C. This procedure yielded a powder or in several cases single crystals suitable for X-ray diffraction. The solid was then washed with pentane and dried under high vacuum for several days to remove residual solvent.

General Procedure 3 (GP 3): A solution of the ligand (100 µmol, 1.0 equiv.) in 5 mL DCM was added to the metal precursor [M]−BF_4 (100 µmol, 1.0 equiv.). The mixture was stirred for 30 minutes, filtered, layered with toluene and pentane and stored at −40 °C. This procedure yielded a powder or in several cases single crystals suitable for X-ray diffraction. The solid was then washed with pentane and dried under high vacuum for several days to remove residual solvent.

Compound [2a-$PdCl_2$]

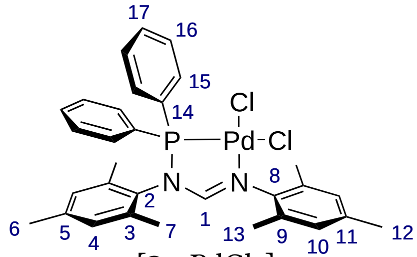

[**2a**-$PdCl_2$]

yield: 350 mg light yellow solid (545 µmol, 78 %, GP 2).

^{1}H-NMR (399.89 MHz, CD_2Cl_2): δ (ppm) = 1.47 (s, 6 H, H-7), 2.23 (s, 3 H, H-6), 2.29 (s, 3 H, H-12), 2.42 (s, 6 H, H-13), 6.75 (s, 2 H, H-4), 6.94 (s, 2 H, H-10), 7.21 (d, J = 37.6 Hz, 1 H, H-1), 7.50–7.59 (m, 4 H, H-16), 7.66–7.75 (m, 2 H, H-17), 7.95–8.07 (m, 4 H, H-15).

^{13}C{^{1}H}-NMR (100.55 MHz, CD_2Cl_2): δ (ppm) = 18.62 (s, 2 C, C-7), 19.46 (s, 2 C, C-13), 21.00 (s, 1 C, C-12), 21.12 (s, 1 C, C-6), 125.51 (d, J = 61.3 Hz, 2 C, C-14), 129.02 (s, 2 C, C-10), 129.35 (d, J = 12.4 Hz, 4 C, C-16), 130.55 (s, 2 C, C-4), 130.97 (d, J = 6.6 Hz, 1-C, C-2), 133.04 (s, 2 C, C-9), 134.16 (d, J = 2.7 Hz, 2 C, C-17), 137.42 (s, 1 C, C-11), 137.66 (s, 2 C, C-3), 135.87 (d, J = 13.5 Hz, 4 C, C-15), 140.77 (s, 1 C, C-5), 141.92 (s, 1 C, C-8), 166.31 (d, J = 19.6 Hz, 1 C, C-1).

^{31}P{^{1}H}-NMR (161.88 MHz, CD_2Cl_2): δ (ppm) = 101.11 (s, 1 P).

EA ($C_{31}H_{33}N_2PPdCl_2$): calcd. C: 58.00 %, H: 5.18 %, N: 4.36 %; found: C: 57.52 %, H: 5.38 %, N: 4.11 %.

MS (LIFDI+): $[M-Cl]^+$ = $C_{31}H_{33}N_2PPdCl^+$ calcd.: 605.1 found: 603.8.

Compound [2a-PdCl]$_2$(BF$_4$)$_2$

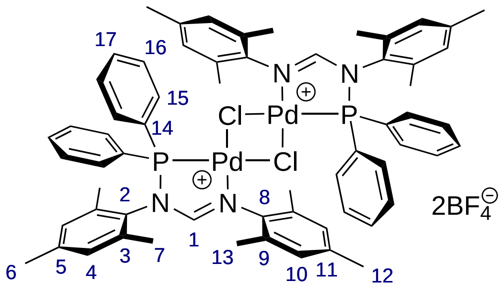

[**2a**-PdCl]$_2$(BF$_4$)$_2$

yield: 81.0 mg colorless solid (58.4 µmol, 75 %, GP 2).

The compound was isolated as a dimer in the solid state. At r.t. a dynamic equilibrium between monomeric and dimeric species was found in $CDCl_3$ solution. Chemical shifts are provided for the average structure in CD_2Cl_2. For a VT-NMR study of compound [**2a**-PdCl]$_2$(BF$_4$)$_2$ see Section 3.

^{1}H-NMR (600.13 MHz, CD_2Cl_2): δ (ppm) = 1.01–2.62 (m, 18 H, H-6/H-7/H-12/H-13), 6.58-6.19 (m, 15 H, H-Ar/H-1).

^{13}C{^{1}H}-NMR (150.90 MHz, CD_2Cl_2): δ (ppm) = 18.94 (br s, 4 C, C-7/C-13), 19.41 (br s, 4 C, C-7/C-13), 21.04 (br s, 2 C, C-6/C-12), 21.15 (br s, 2 C, C-6/C-12), 122.39 (d, J = 65.5 Hz, 4 C, C-14), 128.65 (br s, 2 C, C-Ar), 129.77 (br s, 4 C, C-4/C-10), 130.49 (br s, 8 C, C-15/C-16) 131.02 (br s, 4 C, C-4/C-10), 133.23 (br s, 4 C, C-Ar), 135.36 (br s, 8 C, C-15/C-16), 136.37 (br s, 4 C, C-17), 137.46 (br s, 4 C, C-Ar), 139.61 (br s, 4 C, C-Ar), 142.10 (br s, 2 C, C-Ar), 168.10 (br s, 2 C, C-1).

^{31}P{^{1}H}-NMR (242.93 MHz, CD_2Cl_2): δ (ppm) = 108.85 (br s, 2 P) .

EA ($C_{62}H_{66}Cl_2N_4P_2Pd_2B_2F_8$): calcd. C: 53.71 %, H: 4.80 %, N: 4.04 %; found: C: 53.96 %, H: 4.79 %, N: 4.43 %.

HR-MS (ESI+): $[2M-2BF_4]^{2+}$ = $C_{62}H_{66}Cl_2N_4P_2Pd_2^{2+}$ calcd.: 606.1101 found: 606.1144.

Compound [2a-Pd(allyl)]BF_4

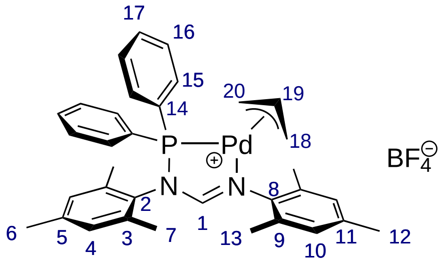

[**2a**-Pd(allyl)]BF_4

yield: 125 mg yellow solid (178 µmol, 83 %, GP 2).

^{1}H-NMR (600.13 MHz, CD_2Cl_2): δ (ppm) = 1.40 (s, 3 H, CH_3), 1.48 (s, 3 H, CH_3), 2.26 (s, 3 H, CH_3), 2.27 (s, 3 H, CH_3), 2.31 (s, 3 H, CH_3), 2.38 (s, 3 H, CH_3), 2.99 (d, J = 12.6 Hz, H-20), 3.83 (dd, J = 10.2 Hz, J = 14.0 Hz, 1 H, H-18), 4.04–4.13 (m, 2 H, H-18/H-20), 5.88–5.97 (m, 1 H, H-19), 6.80 (s, 1 H, H-4/H-10), 6.82 (s, 1 H, H-4/H-10), 6.97 (s, 1 H, H-4/H-10), 6.99 (s, 1 H, H-4/H-10), 7.50 (d, J = 22.3 Hz, 1 H, H-1), 7.50–7.63 (m, 6 H, H-15/H-17), 7.66–7.77 (m, 4 H, H-16).

^{13}C{^{1}H}-NMR (150.90 MHz, CD_2Cl_2): δ (ppm) = 18.49 (s, 1 C, CH_3), 18.58 (s, 1 C, CH_3), 19.20 (s, 2 C, CH_3), 20.93 (s, 1 C, CH_3), 21.04 (s, 1 C, CH_3), 55.79 (d, J = 4.1 Hz, 1 C, C-20), 82.28 (d, J = 31.1 Hz, 1 C, C-18), 123.66 (d, J, 5.9 Hz, 1 C, C-19), 126.80 (s, 1 C, C-Ar), 127.14 (d, J = 19.0 Hz, 1 C, C-Ar), 129.74 (s, 1 C, C-4/C-10), 129.79 (s, 1 C, C-4/C-10), 129.90 (s, 1 C, C-Ar), 129.97 (s, 2 C, C-Ar), 130.03 (s, 1 C, C-Ar), 130.06 (s, 1 C, C-Ar), 130.61 (s, 1 C, C-4/C-10), 130.68 (s, 1 C, C-4/C-10), 132.16 (d, J = 6.7 Hz, 1 C, C-Ar,) 134.32 (s, 2 C, C-Ar), 134.94 (s, 1 C, C-Ar), 135.04 (s, 2 C, C-Ar), 135.16 (s, 2 C, C-Ar), 136.96 (s, 1 C, C-Ar), 137.59 (d, J = 32.3 Hz, 2 C, C-14), 140.79 (s, 1 C, C-Ar), 146.04 (s, 1 C, C-Ar), 166.78 (d, J = 20.3 Hz, 1 C, C-1).

^{31}P{^{1}H}-NMR (242.93 MHz, CD_2Cl_2): δ (ppm) = 98.88 (s, 1 P) .

EA ($C_{34}H_{38}N_2PPdBF_4$ + 0.5 CH_2Cl_2): calcd. C: 55.90 %, H: 5.30 %, N: 3.78 %; found: C: 56.33 %, H: 5.46 %, N: 3.82 %. The presence of half an equivalent of dichloromethane was accounted for.

HR-MS (ESI+): $[M-BF_4]^+$ = $C_{34}H_{38}N_2PPd^+$ calcd.: 613.1806 found: 613.1802.

Compound [2a-Rh(cod)]BF_4

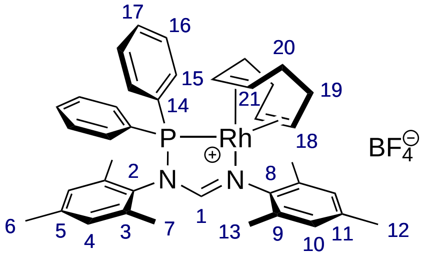

[**2a**-Rh(cod)]BF_4

yield: 137 mg yellow solid (182 µmol, 85 %, GP 3).

^{1}H-NMR (399.89 MHz, CD_2Cl_2): δ (ppm) = 1.34 (s, 6 H, H-7), 2.07 (s, 3 H, H-6), 2.08–2.34 (m, 8 H, H-19/H-20), 2.17 (s, 3 H, H-12), 2.29 (s, 6 H, H-13), 3.58–3.84 (m, 2 H, H-21), 4.46–4.72 (m, 2 H, H-18), 6.57 (s, 2 H, H-4), 6.87 (s, 2 H, H-10), 7.23 (dd, *J* = 2.9 Hz, *J* = 29.2 Hz, 1 H, H-1), 7.40–7.49 (m, 4 H, H-16), 7.52–7.62 (m, 2 H, H-17), 7.63–7.73 (m, 4 H, H-15).

^{13}C{^{1}H}-NMR (100.55 MHz, CD_2Cl_2): δ (ppm) = 19.04 (s, 2 C, C-7), 19.21 (s, 2 C, C-13), 20.91 (s, 1 C, C-6/C-12), 20.99 (s, 1 C, C-6/C-12), 28.95 (s, 2 C, C-20), 31.68 (d, *J* = 2.3 Hz, 2 C, C-19), 83.47 (d, *J* = 11.1 Hz, C-21), 111.36 (dd, *J* = 7.0 Hz, *J* = 10 Hz, C-18), 126.35 (dd, *J* = 48.8 Hz, *J* = 1.9 Hz, 2 C, C-14) 129.41 (d, *J* = 11.2 Hz, 4 C, C-16), 130.18 (s, 2 C, C-4), 130.44 (s, 2 C, C-10), 131.48 (s, 2 C, C-9), 131.68 (d, *J* = 6.8 Hz, 1 C, C-2) 134.04 (d, *J* = 2.1 Hz, 2 C, C-17), 135.38 (d, *J* = 14.8 Hz, 4 C, C-15), 137.34 (s, 2 C, C-3) 137.77 (s, 1 C, C-18), 140.44 (s, 1 C, C-5), 141.64 (s, 1 C, C-11) 169.17 (d, *J* = 20.9 Hz, 1 C, C-1).

^{31}P{^{1}H}-NMR (161.88 MHz, CD_2Cl_2): δ (ppm) = 111.49 (d, *J* = 176.3 Hz, 1 P).

EA ($C_{39}H_{45}N_2PRhBF_4$): calcd. C: 61.43 %, H: 5.95 %, N: 3.67 %; found: C: 62.34 %, H: 6.09 %, N: 3.43 %.

HR-MS (ESI+): $[M{-}BF_4]^+$ = $C_{39}H_{45}N_2PRh^+$ calcd.: 675.2370 found: 675.2366.

Compound [2b-Rh(cod)]BF_4

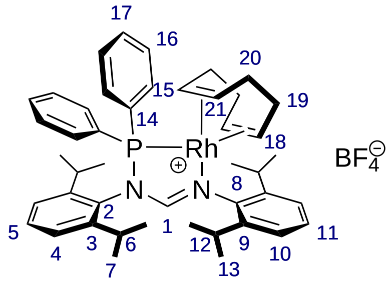

[**2b**-Rh(cod)]BF_4

yield: 115 mg yellow solid (136 µmol, 75 %, GP 3).

^{1}H-NMR (399.89 MHz, CD_2Cl_2): δ (ppm) = 0.21 (d, J = 6.6 Hz, 3 H, H-7), 0.92 (d, J = 6.8 Hz, 3 H, H-7), 1.27 (d, J = 6.9 Hz, 3 H, H-13), 1.61 (d, J = 6.8 Hz, 3 H, H-13), 2.07–2.28 (m, 6 H, H-19/H-20), 2.29–2.41 (m, 2 H, H-19/H-20), 2.68 (sept, J = 6.7 Hz, 2 H, H-6), 3.50 (sept, J = 6.8 Hz, 2 H, H-12), 3.71–3.78 (m, 2 H, H-21), 4.68 (m, 2 H, H-18), 7.06 (d, J = 7.8 Hz, 2 H, H-Ar), 7.28–7.38 (m, 4 H, H-Ar), 7.53–7.70 (m, 11 H, H-Ar/H-1).

^{13}C{^{1}H}-NMR (100.55 MHz, CD_2Cl_2): δ (ppm) = 21.50 (s, 2 C, C-7), 23.13 (s, 2 C, C-7), 26.32 (s, 2 C, C-13), 27.94 (s, 2 C, C-13), 28.46 (d, J = 1.1 Hz, 2 C, C-20), 29.18 (s, 2 C, C-12), 30.08 (s, 2 C, C-6), 31.63 (d, J = 2.3 Hz, 2 C, C-19), 84.25 (d, J = 11.1 Hz, 2 C, C-21), 110.90 (dd, J = 10.0 Hz, J = 6.8 Hz, 2 C, C-18), 125.02 (s, 2 C, C-4/C-10), 125.58 (s, 2 C, C-4/C-10), 126.51 (dd, J = 48.4 Hz, J = 1.9 Hz, 2 C, C-14), 128.77 (s, 1 C, C-5/C-11), 129.93 (d, J = 11.2 Hz, 4 C, C-16), 130.53 (d, J = 6.6 Hz, 1 C, C-2), 131.26 (s, 1 C, C-5/C-11), 134.00 (d, J = 2.1 Hz, 2 C, C-17), 135.38 (d, J = 14.6 Hz, 4 C, C-15), 141.24 (s, 1 C, C-8), 142.33 (s, 2 C, C-3/C-9), 148.25 (s, 2 C, C-3/C-9), 167.24 (d, J = 20.7 Hz, 1 C, C-1).

^{31}P{^{1}H}-NMR (161.88 MHz, CD_2Cl_2): δ (ppm) = 112.23 (d, J = 176.4 Hz, 1 P).

HR-MS (ESI+): $[M-BF_4]^+$ = $C_{45}H_{57}N_2PRh^+$ calcd.: 759.3309 found: 759.3299.

Compound [2c-Rh(cod)]BF$_4$

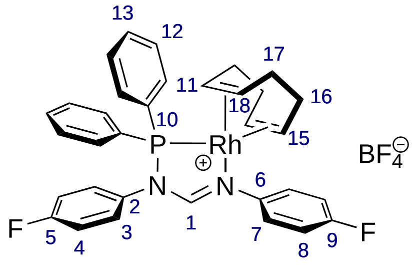

[**2c**-Rh(cod)]BF$_4$

yield: 150 mg yellow solid (212 µmol, 88 %, GP 3).

^{1}H-NMR (399.89 MHz, CD$_2$Cl$_2$): δ (ppm) = 2.11–2.19 (m, 2 H, H-16), 2.19–2.26 (m, 2 H, H-17), 2.26–2.38 (m, 2 H, H-17), 2.38–2.46 (m, 2 H, H-16), 3.71–3.78 (m, 2 H, H-18), 4.99–5.12 (m, 2 H, H-15), 6.72–6.80 (m, 2 H, H-3), 6.83–6.89 (m, 2 H, H-4), 7.11–7.18 (m, 2 H, H-8), 7.28–7.33 (m, 2 H, H-7), 7.58–7.64 (m, 4 H, H-12), 7.66–7.75 (m, 6 H, H-11/H-13), 7.79 (dd, J = 2.7 Hz, J = 27.1 Hz, 1 H, H-1).

^{13}C{^{1}H}-NMR (100.55 MHz, CD$_2$Cl$_2$): δ (ppm) = 28.78 (s, 2 C, C-16), 31.73 (d, J = 2.3 Hz, 2 C, C-17), 81.44 (d, J = 11.8 Hz, 2 C, C-18), 111.03 (dd, J = 10.2 Hz, 2 C, C-15), 116.64 (d, J = 22.9 Hz, 2 C, C-8), 116.89 (d, J = 23.0 Hz, 2 C, C-4), 126.32 (d, J = 8.6 Hz, 2 C, C-7), 126.25 (dd, J = 48.3 Hz, J = 2.0 Hz, 2 C, C-10), 130.10 (d, J = 11.0 Hz, 4 C, C-12), 130.61 (d, J = 9.1 Hz, 2 C, C-3), 132.42 (dd, J = 6.5 Hz, J = 3.4 Hz, 1 C, C-2), 133.68 (d, J = 14.0 Hz, 4 C, C-11), 133.80 (d, J = 2.2 Hz, 2 C, C-13), 143.07 (d, J = 3.0 Hz, 2 C, C-6), 161.80 (d, J = 246.5 Hz, 1 C, C-9), 162.93 (d, J = 250.1 Hz, 1 C, C-5), 167.50 (d, J = 19.2 Hz, 1 C, C-1).

^{31}P{^{1}H}-NMR (242.94 MHz, CD$_2$Cl$_2$): δ (ppm) = 113.41 (d, J = 172.8 Hz, 1).

EA ($C_{33}H_{31}F_2N_2PRhBF_4$): calcd. C: 55.49 %, H: 4.37 %, N: 3.91 %; found: C: 55.43 %, H: 4.51 %, N: 4.09 %.

HR-MS (ESI+): $[M{-}BF_4]^+$ = $C_{33}H_{31}F_2N_2PRh^+$ calcd.: 627.1242 found: 627.1235.

Compound [3a-Rh(cod)]BF_4

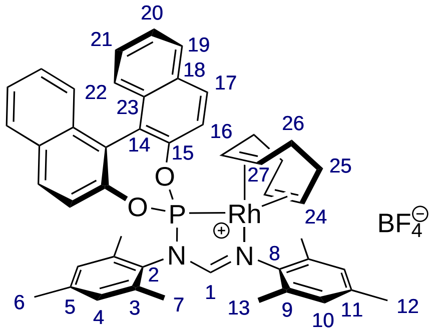

[**3a**-Rh(cod)]BF_4

yield: 222 mg yellow solid (249 µmol, 74 %, GP 3).

^{1}H-NMR (600.13 MHz, CD_2Cl_2): δ (ppm) = 1.99 (s, 3 H, H-6/H-7), 2.06–2.53 (m, 8 H, H-25/H-26), 2.22 (s, 3 H, H-6/H-7), 2.31 (s, 3 H, H-12/H-13), 2.39 (s, 3 H, H-12/H-13), 2.46 (s, 3 H, H-12/H-13), 2.72 (s, 3 H, H-6/H-7), 2.86–2.93 (m, 1 H, H-27), 4.58–4.67 (m, 1 H, H-27), 4.72–4.82 (m, 1 H, H-24), 5.00–5.11 (m, 1 H, H-24), 6.53 (s, 1 H, H-Ar), 6.78 (d, *J* = 9.0 Hz, 1 H, H-Ar), 7.01 (s, 1 H, H-Ar), 7.03 (s, 1 H, H-Ar), 7.10–7.14 (m, 2 H, H-Ar), 7.16–7.27 (m, 3 H, H-Ar), 7.32 (t, *J* = 7.4 Hz, 1 H, H-Ar), 7.46 (t, *J* = 7.6 Hz, 1 H, H-Ar), 7.54–7.59 (m, 2 H, H-Ar), 7.82 (d, *J* = 8.2 Hz, 1 H, H-Ar), 7.91 (d, *J* = 8.9 Hz, 1 H, H-Ar), 8.07 (d, *J* = 8.2 Hz, 1 H, H-Ar), 8.27 (d, *J* = 8.9 Hz, 1 H, H-Ar).

^{13}C{^{1}H}-NMR (150.90 MHz, CD_2Cl_2): δ (ppm) = 18.77 (s, 1 C, C-6/C-7), 18.79 (s, 1 C, C6/C-7), 19.17 (s, 1 C, C-12/C-13), 20.11 (s, 1 C, C-6/C-7), 21.00 (s, 1 C, C-12/C-13), 21.06 (s, 1 C, C-12/C-13), 27.50 (s, 1 C, C-25/C-26), 29.51 (d, *J* = 1.3 Hz, 1 C, C-25/C-26), 31.21 (d, *J* = 2.0 Hz, 1 C, C-25/C-26), 32.89 (d, *J* = 1.9 Hz, 1 C, C-25/C-26), 78.04 (d, *J* = 9.9 Hz, 1 C, C-27), 87.04 (d, *J* = 11.1 Hz, 1 C, C-27), 117.50 (dd, *J* = 13.2 Hz, *J* = 4.2 Hz ,1 C, C-24), 118.20 (dd, *J* = 12.5 Hz, *J* = 5.0 Hz , 1 C, C-24), 119.05 (s, 1 C, C-Ar), 119.90 (d, *J* = 2.2 Hz, 1 C, C-Ar), 121.10 (d, *J* = 2.5 Hz, 1 C, C-Ar), 123.34 (d, *J* = 2.6 Hz, 1 C, C-Ar), 126.41 (s, 1 C, C-Ar) 127.06 (s, 1 C, C-Ar), 127.17 (s, 1 C, C-Ar), 127.21 (s, 1 C, C-Ar), 127.40 (s, 1 C, C-Ar), 127.87 (s, 1 C, C-Ar), 128.59 (d, *J* = 10.9 Hz, 1 C, C-Ar), 128.63 (s, 1 C, C-Ar), 129.22 (s, 1 C, C-Ar), 129.29 (d, *J* = 22.0 Hz, 1 C, C-Ar), 129.97 (d, *J* = 6.6 Hz, 1 C, C-Ar), 130.24 (s, 3 C, C-Ar), 130.37 (s, 1 C, C-Ar), 130.66 (s, 1 C, C-Ar), 130.76 (s, 1 C, C-Ar), 131.59 (s, 1 C, C-Ar), 131.71 (s, 1 C, C-Ar), 131.94 (s, 1 C, C-Ar), 132.17 (s, 1 C, C-Ar), 132.74 (d, *J* = 1.1 Hz, 1 C, C-Ar), 132.50 (s, 1 C, C-Ar), 137.22 (s, 1 C, C-Ar), 137.29 (s, 1 C, C-Ar), 138.15 (s, 1 C, C-Ar), 141.06 (s, 1 C, C-Ar), 141.14 (s, 1 C, C-Ar), 146.05 (d, *J* = 5.6 Hz, 1 C, C-Ar), 148.10 (d, *J* = 16.3 Hz, 1 C, C-Ar), 164.43 (d, *J* = 27.4 Hz, 1 C, C-1).

^{31}P{^{1}H}-NMR (242.94 MHz, CD_2Cl_2): δ (ppm) = 143.62 (d, *J* = 278.5 Hz, 1 P).

EA ($C_{47}H_{47}N_2O_2PRhBF_4$): calcd. C: 63.24 %, H: 5.31 %, N: 3.14 %; found: C: 62.13 %, H: 5.23 %, N: 3.27 %.

HR-MS (ESI+): $[M-BF_4]^+$ = $C_{47}H_{47}N_2O_2PRh^+$ calcd.: 805.2425 found: 805.2419.

Compound [3b-Rh(cod)]BF_4

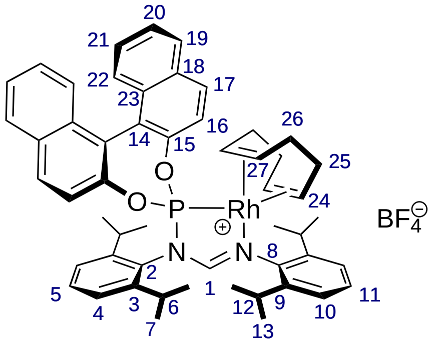

[**3b**-Rh(cod)]BF_4

yield: 130 mg yellow solid (133 µmol, 90 %, GP 3).

^{1}H-NMR (600.13 MHz, CD_2Cl_2): δ (ppm) = 0.00 (d, J = 6.7 Hz, 3 H, H-7/H-13), 0.85 (d, J = 7.0 Hz, 3 H, H-7/H-13), 1.19 (d, J = 6.8 Hz, 3 H, H-7/H-13), 1.30 (d, J = 6.8 Hz, 3 H, H-7), 1.42 (d, J = 6.8 Hz, 3 H, H-7/H-13), 1.45 (d, J = 6.8 Hz, 3 H, H-13), 1.74 (d, J = 6.8 Hz, 3 H, H-7), 1.82 (d, J = 6.7 Hz, 3 H, H-13), 1.94—2.09 (m, 2 H, H-25/H-26), 2.10–2.26 (m, 2 H, H-25/H-26), 2.32–2.37 (m, 2 H, H-25/H-26), 2.38–2.46 (m, 2 H, H-25/H-26), 2.48–2.56 (m, 1 H, H-27), 3.08–3.22 (m, 1 H, H-6/H-12), 3.60 (sept, J = 6.8 Hz, 1 H, H-12), 3.86 (sept, J = 6.8 Hz, 1 H, H-12), 4.64–4.70 (m, 1 H, H-27), 4.89–4.96 (m, 1 H, H-24), 4.98–5.04 (m, 1 H, H-24), 6.19 (d, J = 9.0 Hz, 1 H, H-Ar), 6.94 (d, J = 3.3 Hz, J = 6.0 Hz, 1 H, H-Ar), 7.13 (d, J = 8.5 Hz, 1 H, H-Ar), 7.22 (dd, J = 1.5 Hz, J = 30.8 Hz, 1 H, H-1), 7.24–7.27 (m, 2 H, H-Ar), 7.29–7.39 (m, 4 H, H-Ar), 7.47 (d, J = 8.5 Hz, 1 H, H-Ar), 7.51–7.51 (m, 2 H, H-Ar), 7.58–7.62 (m, 1 H, H-Ar), 7.83 (d, J = 8.2 Hz, 1 H, H-Ar), 7.84 (d, J = 8.8 Hz, 1 H, H-Ar), 8.10 (d, J = 8.2 Hz, 1 H, H-Ar), 8.34 (d, J = 8.9 Hz, 1 H, H-Ar).

^{13}C{^{1}H}-NMR (150.90 MHz, CD_2Cl_2): δ (ppm) = 21.71 (s, 1 C, C-7/C-13), 21.97 (s, 1 C, C-7), 23.28 (s, 1 C, C-7/C-13), 24.33 (s, 1 C, C-13), 25.05 (s, 1 C, C-7), 25.46 (s, 1 C, C-7/C-13), 25.74 (s, 1 C, C-13), 27.18 (s, 1 C, C-7/C-13), 28.29 (s, 1 C, C-6/C-12), 27.37 (s, 1 C, C-25/C-26), 28.47 (s, 1 C, C-6/C-12), 29.14 (d, J = 1.9 Hz, 1 C, C-25/C-26), 30.29 (s, 1 C, C-6), 30.48 (s, 1 C, C-12), 31.29 (d, J = 2.9 Hz, 1 C, C-25/C-26), 33.01 (d, J = 1.7 Hz, 1 C, C-25/C-26), 77.49 (d, J = 10.2, 1 C, C-24), 85.71 (d, J = 11.5 Hz, 1 C, C-24), 116.76 (dd, J = 12.6 Hz, J = 5.4 Hz, 1 C, C-27), 118.35 (s, 1 C, C-Ar), 118.43 (dd, J = 13.4 Hz, J = 4.8 Hz, 1 C, C-27), 120.31 (d, J = 2.6 Hz, 1 C, C-Ar), 120.80 (d, J = 2.4 Hz ,1 C, C-Ar), 123.84 (d, J = 3.4 Hz, 1 C, C-Ar), 124.53 (s, 1 C, C-Ar), 125.32

(s, 1 C, C-Ar), 125.67 (s, 1 C, C-Ar), 126.22 (s, 1 C, C-Ar), 126.56 (s, 1 C, C-Ar), 126.82 (s, 1 C, C-Ar), 127.29 (s, 1 C, C-Ar), 127.33 (s, 1 C, C-Ar), 127.47 (s, 1 C, C-Ar), 128.13 (s, 1 C, C-Ar), 128.62 (s, 1 C, C-Ar), 128.95 (s, 1 C, C-Ar), 129.03 (s, 1 C, C-Ar), 129.25 (s, 1 C, C-Ar), 131.20 (s, 1 C, C-Ar), 131.45 (s, 1 C, C-Ar), 131.92 (s, 1 C, C-Ar), 132.32 (s, 1 C, C-Ar), 132.06 (s, 1 C, C-Ar), 132.45 (d, J = 0.8 Hz, 1 C, C-Ar), 132.67 (d, J = 1.7 Hz, 1 C, C-Ar), 140.25 (s, 1 C, C-Ar), 140.88 (s, 1 C, C-Ar), 142.51 (s, 1 C, C-Ar), 145.73 (d, J = 5.4 Hz, 1 C, C-Ar), 148.41 (d, J = 15.3 Hz, 1 C, C-Ar), 148.42 (s, 1 C, C-Ar), 148.63 (d, J = 1.1 Hz, 1 C, C-Ar), 161.78 (d, J = 25.7 Hz, 1 C, C-1).

^{31}P{^{1}H}-NMR (242.94 MHz, CD_2Cl_2): δ (ppm) = 145.07 (d, J = 273.8 Hz, 1 P).

EA ($C_{53}H_{59}N_2O_2PRhBF_4$): calcd. C: 65.17 %, H: 6.09 %, N: 2.87 %; found: C: 65.40 %, H: 6.37 %, N: 2.89 %.

HR-MS (ESI+): $[M{-}BF_4]^+ = C_{53}H_{59}N_2O_2PRh^+$ calcd.: 889.3364   found: 889.3357.

Compound [3c-Rh(cod)]BF$_4$

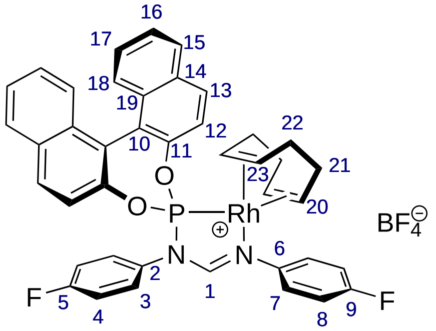

[**3c**-Rh(cod)]BF$_4$

yield: 126 mg yellow solid (148 µmol, 81 %, GP 3).

^{1}H-NMR (399.89 MHz, CD_2Cl_2): δ (ppm) = 2.07–2.29 (m, 4 H, H-21/H-22), 2.34–2.60 (m, 4 H, H-21/H-22), 3.81–3.91 (m, 1 H, H-23), 4.40–4.49 (m, 1 H, H-23), 5.09–5.22 (m, 1 H, H-20), 5.29–5.39 (m, 1 H, H-20), 6.47 (t, J = 8.8 Hz, 1 H, H-Ar), 6.61 (t, J = 8.4 Hz, 2 H, H-Ar), 6.73 (t, J = 8.4 Hz, 1 H, H-Ar), 6.90–6.95 (m ,1 H, H-Ar), 7.03 (d, J = 9.0 Hz, 1 H, H-Ar), 7.16 (d, J = 8.3 Hz, 1 H, H-Ar), 7.17–7.21 (m, 3 H, H-Ar), 7.27 (d, J = 8.2 Hz, 1 H, H-Ar), 7.30–7.32 (m, 2 H, H-Ar), 7.35–7.40 (m, 2 H, H-Ar), 7.46 (t, J = 7.3 Hz, 1 H, H-Ar), 7.57 (d, J = 9.1 Hz, 1 H, H-Ar), 7.83 (d, J 8.2 Hz, 1 H, H-Ar), 7.95 (d, J = 8.6 Hz, 1 H, H-Ar), 8.04 (d, J = 8.2 Hz, 1 H, H-Ar), 8.22 (d, J = 9.0 Hz, 1 H, H-Ar).

^{13}C{^{1}H}-NMR (100.55 MHz, CD_2Cl_2): δ (ppm) = 28.17 (s, 1 C, C-21/C-22), 28.48 (s, 1 C, C-21/C-22), 32.08 (d, J = 2.3 Hz, 1 C, C-21/C-22), 32.13 (d, J = 3.1 Hz, 1 C, C-21/C-22), 77.53 (d, J = 10.5 Hz, 1 C, C-23), 85.95 (d, J = 11.8 Hz, 1 C, C–23), 116.24 (dd, J = 5.7 Hz, J = 12.7 Hz,

1 C, C-20), 116.53 (d, J = 23.1 Hz, 2 C, C-4/C-8), 116.87 (d, J = 23.0 Hz, 2 C, C-4/C-8), 117.37 (dd, J = 5.6 Hz, J = 12.5 Hz, 1 C, C-20), 120.83 (d, J = 2.7 Hz, 1 C, C-Ar), 122.38 (s, 1 C, C-Ar), 122.99 (d, J = 2.5 Hz, 1 C, C-Ar), 126.05 (d, J = 8.6 Hz, 2 C, C-Ar), 126.38 (s, 1 C, C-Ar), 126.82 (s, 1 C, C-Ar), 126.87 (s, 1 C, C-Ar), 127.21 (s, 1 C, C-Ar), 127.37 (s, 1 C, C-Ar), 127.64 (s, 1 C, C-Ar), 128.57 (s, 1 C, C-Ar), 129.09 (s, 1 C, C-Ar), 129.16 (d, J = 9.2 Hz, 1 C, C-Ar), 128.52 (s, 1 C, C-Ar), 129.60 (d, J = 8.8 Hz, 2 C, C-Ar), 130.94 (s, 1 C, C-Ar), 131.27 (dd, J = 2.9 Hz, J = 6.1 Hz, 1 C, C-Ar), 131.92 (s, 1 C, C-Ar), 132.04 (s, 1 C, C-Ar), 132.45 (s, 1 C, C-Ar), 132.73 (s, 1 C, C-Ar), 142.86 (d, J = 3.0 Hz, 1 C, C-Ar), 146.19 (d, J = 5.9 Hz, 1 C, C-Ar), 148.22 (d, J = 14.9 Hz, 1 C, C-Ar), 161.99 (d, J = 247.1 Hz, 1 C, C-9), 162.67 (d, J = 249.8 Hz, 1 C, C-5), 164.15 (d, J = 25.7 Hz, 1 C, C-1).

^{31}P{^{1}H}-NMR (161.88 MHz, CD_2Cl_2): δ (ppm) = 139.10 (d, J = 279.38 Hz, 1 P).[19]

EA ($C_{41}H_{33}F_2N_2O_2PRhBF_4$ + 0.5 C_7H_8): calcd. C: 60.36 %, H: 3.64 %, N: 3.16 %; found: C: 59.51 %, H: 3.99 %, N: 3.43 %. The presence of half an equivalent of toluene was accounted for.

HR-MS (ESI+): $[M{-}BF_4]^+$ = $C_{41}H_{33}F_2N_2O_2PRh^+$ calcd.: 757.1297 found: 757.1294.

Compound [2a-Cp*RhCl]BF_4

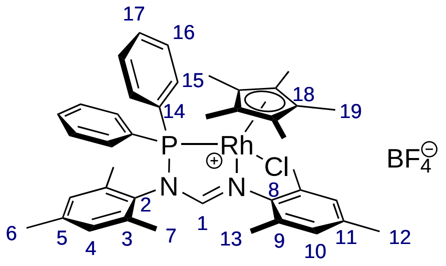

[**2a**-Cp*RhCl]BF_4

yield: 155 mg red-brown solid (188 µmol, 87 %, GP 2).

^{1}H-NMR (600.13 MHz, CD_2Cl_2): δ (ppm) = 1.26 (d, J = 4.0 Hz, H-19), 1.71 (s, 3 H, CH_3), 1.82 (s, 3 H, CH_3), 2.23 (s, 3 H, CH_3), 2.31 (s, 3 H, CH_3), 2.34 (s, 3 H, CH_3), 2.50 (s, 3 H, CH_3), 6.71 (s, 1 H, H-4/H-10), 6.85 (s, 1 H, H-4/H-10), 7.01 (s, 1 H, H-4/H-10), 7.04 (s, 1 H, H-4/H-10), 7.29–7.34 (m, 2 H, H-Ar), 7.37–7.43 (m, 3 H, H-Ar), 7.46–7.53 (m, 2 H, H-Ar), 7.56–7.61 (m, 2 H, H-Ar), 7.64–7.72 (m, 2 H, H-Ar).

^{13}C{^{1}H}-NMR (150.90 MHz, CD_2Cl_2): δ (ppm) = 8.89 (d, J = 1.3 Hz, 5 C, C-19), 20.24 (s, 1 C, CH_3), 20.61 (s, 1 C, CH_3), 20.83 (s, 1 C, CH_3), 20.90 (s, 1 C, CH_3), 20.97 (s, 1 C, CH_3), 21.60 (s, 1 C, CH_3), 102.83 (dd, J = 3.1 Hz, J = 6.5 Hz, 5 C, C-18), 121.90 (d, J = 65.0 Hz, 1 C, C-Ar) 128.42 (d, J = 12.0 Hz, 2 C, C-Ar), 129.34 (d, J = 11.0 Hz, 2 C, C-Ar), 129.70 (s, 1 C, C-Ar), 130.05 (s, 2 C,

C-Ar), 130.79 (s, 1 C, C-Ar), 131.00 (s, 1 C, C-Ar), 131.20 (s, 1 C, C-Ar), 132.59 (s, 1 C, C-Ar), 133.91 (d, J = 2.6 Hz, 1 C, C-Ar), 134.10 (d, J = 2.4 Hz, 1 C, C-Ar), 134.59 (s, 1 C, C-Ar), 134.81 (d, J = 12.2 Hz, 2 C, C-Ar), 135.48 (s, 1 C, C-Ar), 136.35 (d, J = 12.5 Hz, 2 C, C-Ar), 138.00 (s, 1 C, C-Ar), 140.67 (s, 1 C, C-Ar), 141.25 (s, 1 C, C-Ar), 141.72 (s, 1 C, C-Ar), 166.51 (d, J = 19.1 Hz, 1 C, C-1).

^{31}P{^{1}H}-NMR (242.93 MHz, CD_2Cl_2): δ (ppm) = 108.92 (d, J = 152.9 Hz, 1 P).

EA ($C_{41}H_{48}ClN_2PRhBF_4$ + 0.5 CH_2Cl_2): calcd. C: 57.46 %, H: 5.69 %, N: 3.23 %; found: C: 56.79 %, H: 5.83 %, N: 3.21 %. The presence of half an equivalent of dichloromethane was accounted for.

HR-MS (ESI+): $[M-BF_4]^+ = C_{41}H_{48}ClN_2PRh^+$ calcd.: 737.2293 found: 737.2285.

Compound [2a-Ir(cod)]BF_4

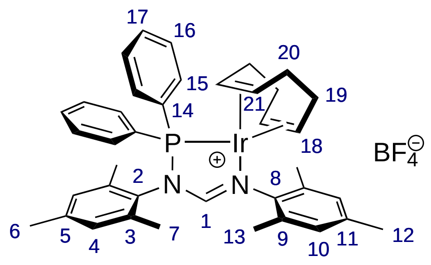

[**2a**-Ir(cod)]BF_4

yield: 163 mg bright red solid (166 µmol, 77 %, GP 2).

^{1}H-NMR (399.89 MHz, CD_2Cl_2): δ (ppm) = 1.50 (s, 6 H, H-7), 2.26 (s, 3 H, H-6), 2.38 (s, 3 H, H-12), 2.44 (s, 6 H, H-13), 3.48–3.59 (m, 2 H, H-21), 4.46–4.65 (m, 2 H, H-18), 6.77 (s, 2 H, H-4), 7.09 (s, 2 H, H-10) , 7.57–7.66 (m, 5 H, H-1/H-16), 7.73–7.79 (m, 2 H, H-17), 7.79–7.87 (m, 4 H, H-15).

^{13}C{^{1}H}-NMR (100.55 MHz, CD_2Cl_2): δ (ppm) = 18.85 (s, 2 C, C-7/C-13), 18.93 (s, 2 C, C-7/C-13), 20.92 (s, 1 C, C-6/C-12), 20.96 (s, 1 C, C-6/C12), 29.55 (d, J = 2.3 Hz, 2 C, C-20), 32.43 (d, J = 2.7 Hz, 2 C, C-19), 68.93 (s, 2 C, C-21), 102.30 (d, J = 12.15, 2 C, C-18), 125.82 (d, J = 58.4 Hz, 2 C, C-14), 129.78 (d, J =11.6 Hz, 4 C, C-16), 130.12 (s, 2 C, C-10), 130.50 (s, 2 C, C-4), 131.33 (d, J = 6.0 Hz, 1 C, C-2) 132.01 (s, 2 C, C-9), 134.35 (d, J = 2.2 Hz, 2 C, C-17), 135.65 (d, J = 14.2 Hz, 4 C, C-15), 137.31 (s, 2 C, C-3), 138.44 (s, 1 C, C-8), 140.78 (s, 1 C, C-11), 140.94 (s, 1 C, C-5), 172.59 (d, J = 18.1 Hz, 1 C, C-1).

^{31}P{^{1}H}-NMR (161.88 MHz, CD_2Cl_2): δ (ppm) = 97.35 (s, 1 P).

EA ($C_{39}H_{45}N_2PIrBF_4$ + CH_2Cl_2): calcd. C: 51.69 %, H: 5.40 %, N: 2.94 %; found: C: 51.33 %,

H: 5.09 %, N: 3.15 %. The presence of one molecule of dichloromethane in the crystal structure was accounted for.

HR-MS (ESI+): $[M{-}BF_4]^+$ = $C_{39}H_{45}N_2PIr^+$ calcd.: 765.2946 found: 765.2940.

Compound [2b-Ir(cod)]BF_4

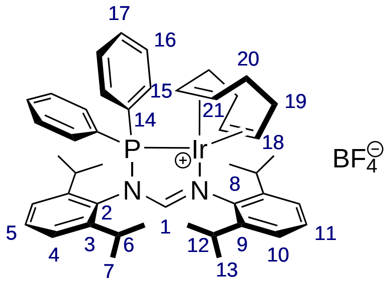

[**2b**-Ir(cod)]BF_4

yield: 136 mg bright red solid (73.0 µmol, 80 %, GP 2).

^{1}H-NMR (600.13 MHz, CD_2Cl_2): δ (ppm) = 0.19 (d, J = 6.6 Hz, 6 H, H-7), 0.91 (d, J = 6.8 Hz, 6 H, H-7), 1.25 (d, J = 6.9 Hz, 6 H, H-13), 1.56 (d, J = 6.8 Hz, 6 H, H-13), 1.92–2.15 (m, 6 H, H-19/H-20), 2.16–2.26 (m, 2 H, H-19/H-20), 2.63 (sept, J = 6.7 Hz, 2 H, H-6), 3.35–3.46 (m, 4 H, H-21/H-12), 4.49–4.56 (m, 2 H, H-18), 7.05–7.11 (m, 2 H, H-Ar), 7.33–7.39 (m, 4 H, H-Ar), 7.53–7.59 (m, 4 H, H-16), 7.59–7.65 (m, 4 H, H-15), 7.65–7.69 (m, 2 H, H-17), 7.78 (d, J = 25.3 Hz, 1 H, H-1).

^{13}C{^{1}H}-NMR (100.55 MHz, CD_2Cl_2): δ (ppm) = 21.43 (s, 2 C, C-7), 23.24 (s, 2 C, C-13), 26.44 (s, 2 C, C-13), 28.01 (s, 2 C, C-7), 29.02 (s, 2 C, C-12), 29.09 (d, J = 1.9 Hz, 2 C, C-17), 30.07 (s, 2 C, C-6) 32.35 (d, J = 2.7 Hz, 2 C, C-16), 69.87 (s, 2 C, C-21), 101.77 (d, J = 12.1 Hz, 2 C, C-18), 124.97 (s, 2 C, C-4/C-10), 125.62 (s, 2 C, C-4/C-10), 125.86 (d, J = 58.3 Hz, 2 C, C-14), 129.36 (s, 1 C, C-5/C-11), 129.89 (d, J = 11.6 Hz, 4 C, C-16), 130.03 (d, J = 5.6 Hz, 1 C, C-2), 131.45 (s, 1 C, C5/C-11), 134.26 (d, J = 2.2 Hz, 2 C, C-17), 140.34 (s, 1 C, C-8), 142.78 (s, 2 C, C-3/C-9), 148.09 (s, 2 C, C-3/C-9), 135.61 (d, J = 14.0 Hz, 4 C, C-15), 170.56 (d, J = 18.2 Hz, 1 C, C-1).

^{31}P{^{1}H}-NMR (242.94 MHz, CD_2Cl_2): δ (ppm) = 100.93 (s, 1 P).

EA ($C_{45}H_{47}N_2PIrBF_4$): calcd. C: 57.75 %, H: 6.14 %, N: 2.99 %; found: C: 57.62 %, H: 6.02 %, N: 2.92 %.

HR-MS (ESI+): $[M{-}BF_4]^+$ = $C_{45}H_{47}N_2PIr^+$ calcd.: 849.3886 found: 849.3883.

Compound [2c-Ir(cod)]BF_4

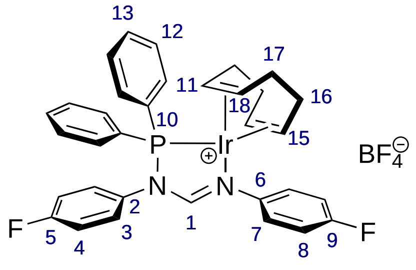

[**2c**-Ir(cod)]BF_4

yield: 170 mg red solid (212 µmol, 88 %, GP 2).

^{1}H-NMR (600.13 MHz, CD_2Cl_2): δ (ppm) = 1.97–2.05 (m, 2 H, H-16), 2.05–2.11 (m, 2 H, H-17), 2.14–2.21 (m, 2 H, H-17), 2.21–2.30 (m, 2 H, H-16), 3.41–3.49 (m, 2 H, H-18), 4.81–4.90 (m, 2 H, H-15), 6.76–6.81 (m, 2 H, H-3), 6.84–6.91 (m, 2 H, H-4), 7.16–7.21 (m, 2 H, H-8), 7.31–7.36 (m, 2 H, H-7), 7.58–7.63 (m, 4 H, H-12), 7.67–7.73 (m, 6 H, H-11/H-13), 8.03 (d, *J* = 23.9 Hz, 1 H, H-1).

^{13}C{^{1}H}-NMR (150.90 MHz, CD_2Cl_2): δ (ppm) = 29.50 (d, *J* = 2.0 Hz, 2 C, C-16), 32.47 (d, *J* = 2.9 Hz, 2 C, C-17), 67.02 (s, 2 C, C-18), 101.75 (d, *J* = 12.3 Hz, 2 C, C-15), 116.60 (d, *J* = 22.8 Hz, 2 C, C-8), 116.98 (d, *J* = 23.1 Hz, 2 C, C-4), 126.04 (d, *J* = 58.0 Hz, 2 C, C-10), 127.03 (d, *J* = 8.8 Hz, 2 C, C-7), 130.06 (d, *J* = 11.3 Hz, 4 C, C-12), 130.69 (d, *J* = 8.8 Hz, 2 C, C-3), 132.03 (d, *J* = 5.1 Hz, *J* = 3.2 Hz, 1 C, C-2), 133.90 (d, *J* = 13.8 Hz, 4 C, C-11), 134.09 (d, *J* = 2.2 Hz, 2 C, C-13), 142.10 (d, *J* = 3.3 Hz, 1 C, C-6), 162.20 (d, *J* = 247.3 Hz, 1 C, C-9), 163.13 (d, *J* = 250.6 Hz, 1 C, C-5), 170.67 (d, *J* = 17.1 Hz, 1 C, C-1).

^{31}P{^{1}H}-NMR (242.94 MHz, CD_2Cl_2): δ (ppm) = 99.91 (s, 1 P).

EA ($C_{33}H_{31}F_2N_2PIrBF_4$): calcd. C: 49.32 %, H: 3.89 %, N: 3.49 %; found: C: 50.37 %, H: 4.14 %, N: 3.68 %.

HR-MS (ESI+): $[M{-}BF_4]^+$ = $C_{33}H_{31}F_2N_2PIr^+$ calcd.: 717.1817 found: 717.1807.

Compound [3a-Ir(cod)]BF_4

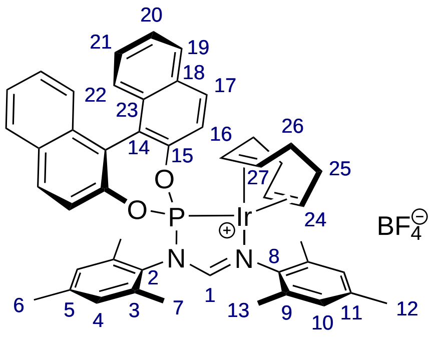

[**3a**-Ir(cod)]BF_4

yield: 160 mg brown-red solid (163 µmol, 65 %, GP 2).

^{1}H-NMR (399.89 MHz, CD_2Cl_2): δ (ppm) = 1.94–2.34 (m, 8 H, H-26/H-27), 2.05 (s, 3 H, H-6/H-7), 2.21 (s, 3 H, H-6/H-7), 2.35 (s, 3 H, H-12/H-13), 2.39 (s, 3 H, H-12/H-13), 2.45 (s, 3 H, H-12/H-13), 2.53–2.64 (m, 1 H, H-27), 2.70 (s, 3 H, H-6/H-7), 4.10–4.27 (m, 1 H, H-27), 4.65–4.79 (m, 1 H, H-24), 5.04–5.19 (m, 1 H, H-24), 6.55 (s, 1 H, H-Ar), 6.89 (d, *J* = 9.0 Hz, 1 H, H-Ar), 7.02–7.14 (m, 4 H, H-Ar), 7.18 (d, *J* = 8.6 Hz, 1 H, H-Ar), 7.21–7.27 (m, 1 H, H-Ar), 7.32 (d, *J* = 29.5 Hz, 1 H, H-1), 7.29–7.35 (m, 1 H, H-Ar), 7.42–7.49 (m, 1 H, H-Ar), 7.53–7.65 (m, 2 H, H-Ar), 7.84 (t, *J* = 8.7 Hz, 2 H, H-Ar), 8.07 (d, *J* = 8.2 Hz, 1 H, H-Ar), 8.24 (d, *J* = 8.9 Hz, 1 H, H-Ar).

^{13}C{^{1}H}-NMR (100.55 MHz, CD_2Cl_2): δ (ppm) = 18.51 (s, 1 C, C-12/C-13), 18.78 (s, 1 C, C-6/C-7), 18.94 (s, 1 C, C-12/C-13), 20.01 (s, 1 C, C-6/C-7), 20.96 (s, 1 C, C-6/C-7), 20.97 (s, 1 C, C-12/C-13), 28.07 (s, 1 C, C-25/C-26), 30.45 (s, 1 C, C-25/C-26), 32.11 (s, 1 C, C-25/C-26), 33.59 (s, 1 C, C-25/C-26), 64.30 (s, 1 C, C-27), 72.60 (s, 1 C, C-27), 111.03 (d, *J* = 16.0 Hz, 1 C, C-24), 112.19 (d, *J* = 15.3 Hz, 1 C, C-24), 119.14 (d, *J* = 1.8 Hz, 1 C, C-Ar), 119.81 (d, *J* = 2.6 Hz, 1 C, C-Ar), 121.17 (d, *J* = 2.8 Hz, 1 C, C-Ar), 123.04 (d, *J* = 3.2 Hz, 1 C, C-Ar), 126.40 (s, 1 C, C-Ar), 127.01 (s, 1 C, C-Ar), 127.20 (s, 2 C, C-Ar), 127.41 (s, 1 C, C-Ar), 127.81 (s, 1 C, C-Ar), 128.61 (s, 1 C, C-Ar), 129.20 (s, 1 C, C-Ar), 129.61 (d, *J* = 5.6 Hz, 1 C, C-Ar), 130.14 (s, 1 C, C-Ar), 130.18 (s, 1 C, C-Ar), 130.23 (s, 1 C, C-Ar), 130.65 (s, 1 C, C-Ar), 130.89 (s, 1 C, C-Ar), 131.04 (s, 1 C, C-Ar), 131.61 (s, 1 C, C-Ar), 131.91 (d, *J* = 1.1 Hz, 1 C, C-Ar), 132.09 (s, 1 C, C-Ar), 132.27 (s, 1 C, C-Ar), 132.45 (d, *J* = 1.2 Hz, 1 C, C-Ar), 132.67 (d, *J* = 1.7 Hz, 1 C, C-Ar), 137.07 (s, 2 C, C-Ar), 138.80 (s, 1 C, C-Ar), 140.67 (s, 1 C, C-Ar), 141.18 (s, 1 C, C-Ar), 146.01 (d, *J* = 6.1 Hz, 1 C, C-Ar), 147.85 (d, *J* = 15.5 Hz, 1 C, C-Ar), 167.98 (d, *J* = 24.0 Hz, 1 C, C-1).

^{31}P{^{1}H}-NMR (161.88 MHz, CD_2Cl_2): δ (ppm) = 125.08 (s, 1 P).

EA ($C_{47}H_{47}N_2O_2PIrBF_4$): calcd. C: 57.49 %, H: 4.85 %, N: 2.85 %; found: C: 57.27 %, H: 4.84 %, N: 2.99 %.

HR-MS (ESI+): $[M-BF_4]^+$ = $C_{47}H_{47}N_2O_2PIr^+$ calcd.: 895.2999 found: 895.2998.

Compound [3b-Ir(cod)]BF_4

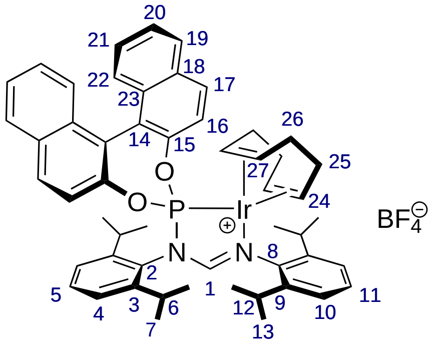

[**3b**-Ir(cod)]BF_4

yield: 127 mg dark red solid (119 µmol, 81 %, GP 2).

^{1}H-NMR (600.13 MHz, CD_2Cl_2): δ (ppm) = 0.05 (d, J = 6.7 H, 3 H, H-13), 0.87 (d, J = 7.0 H, 3 H, H-13), 1.20 (d, J = 6.8 H, 3 H, H-7), 1.30 (d, J = 6.8 H, 3 H, H-7), 1.40−1.44 (m, 6 H, H-7/H-13), 1.68 (d, J = 6.8 H, 3 H, H-7), 1.81 (d, J = 6.7 H, 3 H, H-13), 1.91−2.27 (m, 9 H, H-25/H-26/H-27), 3.11 (sept, J = 6.8 Hz, 1 H, H-6), 3.17 (sept, J = 6.9 Hz, 1 H, H-12), 3.52 (sept, J = 6.8 Hz, 1 H, H-12), 3.76 (sept, J = 6.4 Hz, 1 H, H-6), 4.19−4.25 (m, 1 H, H-27), 4.91−5.00 (m, 2 H, H-24), 6.28 (d, J = 9.1 Hz, 1 H, H-Ar), 6.91 (dd, J = 2.1 Hz, J = 7.1 Hz, 1 H, H-Ar), 7.12 (d, J = 8.7 Hz 1 H, H-Ar), 7.21−7.30 (m 3 H, H-Ar), 7.32−7.39 (m, 4 H, H-Ar), 7.44−7.54 (m, 4 H, H-Ar), 7.57−7.62 (m 1 H, H-Ar), 7.76 (d, J = 8.9 Hz, 1 H, H-Ar), 7.82 (d, J = 7.8 Hz, 1 H, H-Ar), 8.09 (d, J = 8.1 Hz, 1 H, H-Ar), 8.29 (d, J = 8.9 Hz, 1 H, H-Ar).

^{13}C{^{1}H}-NMR (150.90 MHz, CD_2Cl_2): δ (ppm) = 21.91 (s, 1 C, C-13), 22.02 (s, 1 C, C-7), 23.40 (s, 1 C, C-7/C-13), 24.34 (s, 1 C, C-13), 25.17 (s, 1 C, C-7), 25.52 (s, 1 C, C-13), 25.87 (s, 1 C, C-7/C-13), 27.26 (s, 1 C, C-7), 28.19 (s, 1 C, C-6), 28.32 (d, J = 2.3 Hz, 1 C, C-25/C-26), 28.57 (s, 1 C, C-12), 29.87 (d, J = 3.0 Hz, 1 C, C-25/C-26) 30.21 (s, 1 C, C-12), 30.53 (s, 1 C, C-6), 32.40 (d, J = 3.9 Hz, 1 C, C-25/C-26), 33.44 (d, J = 3.8 Hz, 1 C, C-25/C-26), 63.68 (s, 1 C, C-27), 71.54 (s, 1 C, C-27), 110.14 (d, J = 15.4 Hz, 1 C, C-24), 117.73 (d, J = 16.1 Hz, 1 C, C-24), 118.44 (s, 1 C, C-Ar), 120.25 (d, J = 2.4 Hz, 1 C, C-Ar), 120.91 (d, J = 2.4 Hz, 1 C, C-Ar), 123.59 (d, J = 3.4 Hz, 1 C, C-Ar), 124.55 (s, 1 C, C-Ar), 125.36 (s, 1 C, C-Ar), 125.68 (s, 1 C, C-Ar), 126.27 (s, 1 C, C-Ar), 126.59 (s, 1 C, C-Ar), 126.86 (s, 1 C, C-Ar), 127.28 (s, 1 C, C-Ar), 127.36 (s, 1 C, C-Ar), 127.50 (s, 1 C, C-Ar), 128.12 (s, 1 C, C-Ar), 128.63 (s, 1 C, C-Ar), 128.69 (d, J = 4.9 Hz, 1 C, C-Ar), 129.24

(s, 1 C, C-Ar), 129.66 (s, 1 C, C-Ar), 131.28 (s, 1 C, C-Ar), 131.45 (s, 1 C, C-Ar), 132.00 (s, 1 C, C-Ar), 132.04 (s, 1 C, C-Ar), 132.21 (s, 1 C, C-Ar), 132.41 (d, *J* = 1.0 Hz, 1 C, C-Ar), 132.61 (d, *J* = 1.8 Hz, 1 C, C-Ar), 139.88 (s, 1 C, C-Ar), 141.45 (s, 1 C, C-Ar), 143.14 (s, 1 C, C-Ar), 145.76 (d, *J* = 5.8 Hz, 1 C, C-Ar), 148.09 (d, *J* = 15.5 Hz, 1 C, C-Ar), 148.35 (s, 1 C, C-Ar), 148.46 (s, 1 C, C-Ar), 164.89 (d, *J* = 22.6 Hz, 1 C, C-1).

$^{31}P\{^1H\}$-NMR (242.93 MHz, CD_2Cl_2): δ (ppm) = 127.52 (s, 1 P) .

EA ($C_{53}H_{59}N_2O_2PIrBF_4$): calcd. C: 59.71 %, H: 5.58 %, N: 2.63 %; found: C: 59.78 %, H: 5.81 %, N: 2.74 %.

HR-MS (ESI+): $[M{-}BF_4]^+$ = $C_{53}H_{59}N_2O_2PIr^+$ calcd.: 979.3938 found: 979.3923.

Compound [3c-Ir(cod)]BF_4

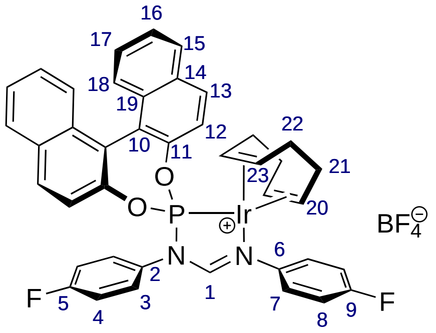

[**3c**-Ir(cod)]BF_4

yield: 140 mg dark red solid (150 µmol, 82 %, GP 2).

^1H-NMR (600.13 MHz, $CDCl_2$): δ (ppm) = 1.47−1.64 (m, 2 H, H-21/H-22), 1.85−2.23 (m, 6 H, H-21/H-22/H-23), 3.42−3.60 (m, 1 H, H-23), 3.83−4.01 (m, 1 H, H-23), 5.01−5.15 (m, 1 H, H-20), 5.32−5.36 (m, 1 H, H-20) 6.45−6.58 (m, 2 H, H-Ar), 6.96−6.98 (m, 1 H, H-Ar), 7.06−7.10 (m, 2 H, H-Ar), 7.13−7.17 (m, 4 H, H-Ar), 7.11−7.26 (m, 4 H, H-Ar), 7.38−7.41 (m, 1 H, H-Ar), 7.46−7.49 (m, 1 H, H-Ar), 7.50 (d, *J* = 9.0 Hz, 1 H, H-Ar), 7.68 (d, *J* = 29.6 Hz, 1 H, H-1), 7.75 (d, *J* = 8.0 Hz, 1 H, H-Ar) 7.80 (d, *J* = 8.9 Hz, 1 H, H-Ar) 7.96 (d, *J* = 8.2 Hz, 1 H, H-Ar), 8.12 (d, *J* = 10.1 Hz, 1 H, H-Ar).

$^{13}C\{^1H\}$-NMR (150.90 MHz, CD_2Cl_2): δ (ppm) = 29.17 (s, 2 C, C-21/C-22), 32.75 (s, 1 C, C-21/C-22), 33.30 (s, 1 C, C-21/C-22), 64.16 (s, 1 C, C-23), 71.51 (s, 1 C, C-23), 109.31 (d, *J* = 13.3 Hz, 1 C, C-20), 110.92 (d, *J* = 13.9 Hz, 1 C, C-20), 116.64 (d, *J* = 23.2 Hz, 2 C, C-4/C-8), 116.88 (d, *J* = 23.1 Hz, 2 C, C-4/C-8), 120.45 (s, 2 C, C-Ar), 120.79 (d, *J* = 3.0 Hz, 2 C, C-Ar), 125.60 (s, 1 C, C-Ar), 126.47 (s, 1 C, C-Ar), 126.73 (d, *J* = 8.6 Hz, 2 C, C-3/C-7), 126.89 (s, 2 C, C-Ar), 127.29

(s, 1 C, C-Ar), 127.42 (s, 1 C, C-Ar), 127.69 (s, 1 C, C-Ar), 128.53 (s, 1 C, C-Ar), 128.61 (s, 1 C, C-Ar), 129.09 (s, 1 C, C-Ar), 129.34 (s, 1 C, C-Ar), 129.51 (d, *J* = 9.1 Hz, 2 C, C-3/C-7), 131.08 (s, 1 C, C-Ar) 131.64 (s, 1 C, C-Ar), 131.89 (s, 1 C, C-Ar), 132.02 (s, 1 C, C-Ar), 132.46 (s, 1 C, C-Ar), 145.96 (d, *J* = 8.8 Hz, 1 C, C-Ar), 147.91 (d, *J* = 14.5 Hz, 1 C, C-Ar), 162.40 (d, *J* = 247.9 Hz, 1 C, C-9), 162.78 (d, *J* = 250.1 Hz, 1 C, C-5), 167.34 (d, *J* = 23.7 Hz, 1 C, C-1).

^{31}P{^{1}H}-NMR (242.95 MHz, CD_2Cl_2): *δ* (ppm) = 122.49 (s, 1 P).[19]

EA ($C_{41}H_{33}F_2N_2O_2PIrBF_4$ + 0.5 C_7H_8): calcd. C: 54.55 %, H: 3.81 %, N: 2.86 %; found: C: 54.78 %, H: 3.91 %, N: 2.88 %. The presence of half an equivalent of toluene was accounted for.

HR-MS (ESI+): $[M-BF_4]^+$ = $C_{41}H_{33}F_2N_2O_2PIr^+$ calcd.: 847.1871 found: 847.1859.

Compound [2a-Cp*IrI]BF_4

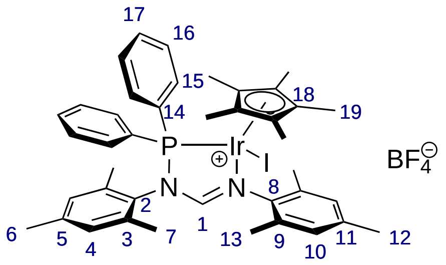

[**2a**-Cp*IrI]BF_4

yield: 165 mg orange solid (164 µmol, 76 %, GP 2).

^{1}H-NMR (600.13 MHz, CD_2Cl_2): *δ* (ppm) = 1.41 (d, *J* = 2.3 Hz, 18 H, H-19), 1.50 (s, 3 H, H-6/H-7), 2.05 (s, 3 H, H-6/H-7), 2.21 (s, 3 H, H-6/H-7), 2.23 (s, 3 H, H-12/H-13), 2.34 (s, 3 H, H-12/H-13), 2.42 (s, 3 H, H-12/H-13), 6.77 (s, 1 H, H-4/H-10), 6.80 (s, 1 H, H-4/H-10), 6.87 (s, 1 H, H-4/H-10), 6.96 (s, 1 H, H-4/H-10), 7.09 (d, *J* = 21.2 Hz, 1 H, H-1), 7.11–7.18 (m, 2 H, H-Ar), 7.26–7.35 (m, 4 H, H-Ar), 7.42–7.50 (m, 2 H, H-Ar), 7.51–7.56 (m, 1 H, H-Ar), 7.56–7.61 (m, 1 H, H-Ar).

^{13}C{^{1}H}-NMR (150.90 MHz, CD_2Cl_2): *δ* (ppm) = 9.51 (d, *J* = 1.0 Hz, 5 C, C-19), 19.94 (s, 1 C, C-6/C-7), 20.73 (s, 1 C, C-6/C-7), 20.83 (s, 1 C, CH_3), 20.91 (s, 1 C, CH_3), 22.74 (s, 1 C, CH_3), 25.67 (s, 1 C, C-12/C-13), 97.25 (d, *J* = 3.0 Hz, 5 C, C-18), 127.05 (d, *J* = 78.4 Hz, 1 C, C-14), 128.33 (d, *J* = 12.8 Hz, 2 C, C-Ar), 129.34 (d, *J* = 10.9 Hz, 2 C, C-Ar), 129.97 (s, 1 C, C-Ar), 130.85 (s, 1 C, C-Ar), 131.25 (s, 1 C, C-Ar), 131.35 (s, 1 C, C-Ar), 132.85 (s, 1 C, C-Ar), 133.06 (d, *J* = 6.4 Hz, 1 C, C-Ar), 133.82 (d, *J* = 51.2 Hz, 1 C, C-14), 134.22 (s, 1 C, C-Ar), 134.43 (d, *J* = 2.7 Hz, 1 C, C-Ar), 134.36 (d, *J* = 2.7 Hz, 1 C, C-Ar), 135.39 (br s, 2 C, C-Ar), 135.53 (d,

J = 12.9 Hz, 2 C, C-Ar), 135.74 (s, 1 C, C-Ar), 138.59 (s, 1 C, C-Ar), 140.92–140.97 (m, 2 C, C-Ar), 141.81 (d, J = 0.6 Hz, C-Ar), 168.37 (d, J = 16.6 Hz, 1 C, C-1).

$^{31}P\{^{1}H\}$-NMR (242.93 MHz, CD_2Cl_2): δ (ppm) = 80.64 (s, 1 P).

EA ($C_{41}H_{48}N_2PIIrBF_4$): calcd. C: 49.27 %, H: 5.32 %, N: 2.74 %; found: C: 49.76 %, H: 4.96 %, N: 2.72 %.

HR-MS (ESI+): $[M{-}BF_4]^+$= $C_{41}H_{48}N_2PIIr^+$ calcd.: 919.2229 found: 919.2227.

Compound [5-Pd(2-Me-allyl)]OTf

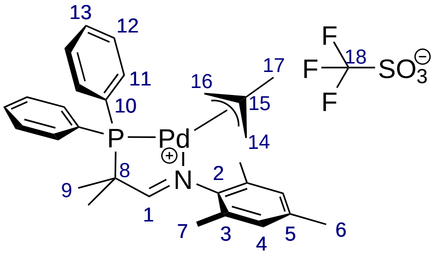

[**5**-Pd(2-Me-allyl)]OTf

yield: 287 mg yellow solid (536 µmol, 41 %, GP 2).

^{1}H-NMR (600.13 MHz, $CDCl_3$): δ (ppm) = 1.52 (d, J = 3.2 Hz, 3 H, H-9), 1.55 (d, J = 3.2 Hz, 3 H, H-9), 1.87 (s, 3 H, H-6/H-17), 2.14 (s, 6 H, H-7), 2.27 (s, 3 H, H-6/H-17), 3.20 (br s, 2 H, H-14/H-16), 3.51 (br s, 1 H, H-14/H-16), 3.63 (br s, 1 H, H-14/H-16), 6.87 (s, 1 H, H-4), 7.53–7.61 (m, 6 H, H-Ar), 7.62–7.70 (m, 4 H, H-Ar), 8.30 (d, J = 19.4 Hz), 1 H, H-Ar).

^{13}C-NMR (150.90 MHz, $CDCl_3$): δ (ppm) = 18.93 (s, 2 C, C-7), 20.84 (s, 1 C, C-6/C-17), 23.69 (br s, 2 C, C-9), 23.98 (s, 1 C, C-6/C-17), 50.60 (d, J = 23.4 Hz, 1 C, C-8), 54.55 (s, 1 C, C-16), 80.22 (d, J = 30.1 Hz, 1 C, C-14), 120.95 (q, J = 321.0 Hz, 1 C, C-18), 126.80 (d, J = 41.8 Hz, 2 C, C-10), 127.13 (s, 2 C, C-3) 129.52 (s, 2 C, C-4), 129.62 (d, J = 10.5 Hz, 4 C, C-12), 132.24 (d, J = 2.1 Hz, 2 C, C-13), 134.03 (d, J = 12.3 Hz, 4 C, C-11), 136.42 (s, 1 C, C-5), 136.98 (d, J = 5.4 Hz, 1 C, C-15), 148.56 (s, 1 C, C-2), 185.71 (s, 1 C, C-1).

^{31}P-NMR (242.94 MHz, $CDCl_3$): δ (ppm) = 58.79 (br s, 1 P).

HR-MS (ESI$^+$): [M−OTf] = $C_{29}H_{35}NPPd^+$ calcd.: 534.1536 found: 534.1539.

Compound [5-Rh(cod)]BF_4

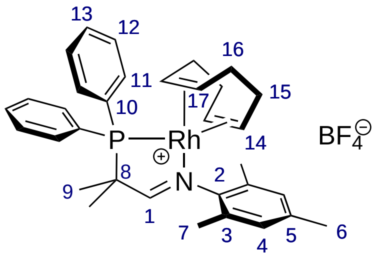

[**5**-Rh(cod)]BF_4

yield: 232 mg orange solid (398 µmol, 52 %, GP 3).

^{1}H-NMR (600.13 MHz, $CDCl_3$): δ (ppm) = 1.52 (d, J = 12.1 Hz, 6 H, H-9), 1.98–2.05 (m, 2 H, H-15/H-16), 2.08–2.14 (m, 2 H, H-15/H-16), 2.18–2.34 (m, 4 H, H-15/H-16), 2.20 (s, 6 H, H-7), 2.22 (s, 3 H, H-6), 4.16 (br s, 2 H, H-17), 4.28 (br s, 2 H, H-14), 6.86 (s, 2 H, H-4), 7.56-7.61 (m, 6 H, H-Ar), 7.62-7.65 (m, 4 H, H-Ar), 8.12-8.17 (dd, J = 27.2 Hz, J = 3.0 Hz, 1 H, H-1).

^{13}C-NMR (150.90 MHz, $CDCl_3$): δ (ppm) = 19.14 (s, 2 C, C-7), 20.88 (s, 1 C, C-6), 24.24 (s, 2 C, C-9), 28.62 (d, J = 1.0 Hz, 2 C, C-16), 31.59 (d, J = 2.5 Hz, 2 C, C-15), 50.89 (d, J = 23.5 Hz, 1 C, C-8), 80.72 (d, J = 17.0 Hz, 2 C, C-17), 106.60 (dd, J = 2.6 Hz, J = 9.8 Hz, 2 C, C-14), 125.12 (d, J = 40.1 Hz, 2 C, C-10), 128.62 (s, 2 C, C-3), 129.46 (d, J = 9.9 Hz, 4 C, C-12), 129.85 (s, 2 C, C-4), 132.09 (d, J = 2.3 Hz, 2 C, C-13), 134.03 (d, J = 10.5 Hz, 4 C, C-11), 137.32 (s, 1 C, C-5), 143.98 (s, 1 C, C-2), 190.28 (d, J = 12.3 Hz, 1 C, C-1).

^{31}P-NMR (242.94 MHz, $CDCl_3$): δ (ppm) = 66.66 (d, J = 154.9 Hz, 1 P).

EA ($C_{34}H_{40}F_3NO_3PSRh$): calcd. C: 59.04 %, H: 6.01 %, N: 2.09 %; found: C: 59.52 %, H: 5.98 %, N: 2.04 %.

HR-MS (ESI^{+}): [M–OTf] = $C_{33}H_{40}NPRh^{+}$ calcd: 584.1948 found: 594.1947.

Compound [5-Ir(cod)]OTf

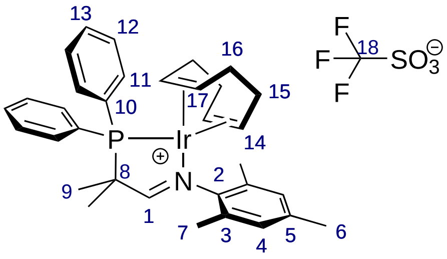

[**5**-Ir(cod)]OTf

yield: 180 mg red crystalline solid (269.1 µmol, 67 %, GP 2).

^{1}H-NMR (600.13 MHz, CDCl$_3$): δ (ppm) = 1.55 (d, J = 12.0 Hz, 6 H, H-9), 1.69 (br s, 4 H, H-15/H-16), 2.08 (d,J = 12.1 Hz, 4 H, H-15/H-16), 2.20 (s, 6 H, H-7), 2.27 (s, 3 H, H-6), 3.96 (br s, 4 H, H-14/H-17), 6.90 (s, 2 H, H-4), 7.57–7.62 (m, 6 H, H-Ar), 7.63–7.68 (m, 4 H, H-Ar), 8.66 (d, J = 2.4 Hz, 1 H, H-1).

^{13}C-NMR (150.90 MHz, CDCl$_3$): δ (ppm) = 19.03 (s, 2 C, C-7), 20.91 (s, 1 C, C-6), 24.16 (s, 2 C, C-9), 29.47 (br s, 2 C, C-15/C-16), 32.30 (br s, 2 C, C-15/C-16), 51.72 (d, J = 29.3 Hz, 1 C, C-8), 66.88 (br s, 2 C, C-14/C-17), 95.45 (br s, 2 C, C-14/C-17), 120.86 (q, J = 320.7 Hz, 1 C, C-18), 124.43 (d, J = 48.8 Hz, 2 C, C-10), 129.19 (s, 2 C, C-3), 129.55 (d, J = 10.3 Hz, 4 C, C-12), 129.78 (s, 2 C, C-4), 132.34 (d, J = 2.4 Hz, 2 C, C-13), 134.25 (d, J = 10.4 Hz, 4 C, C-11), 138.27 (s, 1 C, C-5), 143.47 (s, 1 C, C-2), 195.45 (d, J = 10.1 Hz, 1 C, C-1).

^{31}P-NMR (242.94 MHz, CDCl$_3$): δ (ppm) = 55.30 ppm (s, 1 P).

EA ($C_{34}H_{40}F_3NO_3PSIr$): calcd. C: 49.62 %, H: 4.90 %, N: 1.70 %; found: C: 48.99 %, H: 4.93 %, N: 1.79 %.

HR-MS (ESI$^+$): [M−OTf]$^+$ = $C_{33}H_{40}IrNP^+$ calcd.: 674.2522 found: 674.2513.

Compound [5-Cp*IrI]OTf

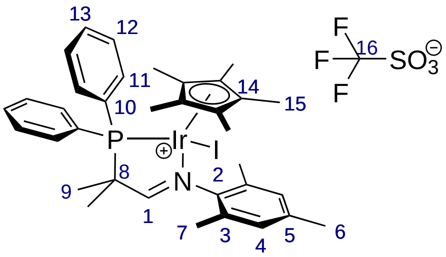

[**5**-Cp*IrI]OTf

yield: 51.0 mg orange solid (72.8 µmol, 80 %, GP 2).

^{1}H-NMR (600.13 MHz, $CDCl_3$): δ (ppm) = 1.34 (d, J = 2.3 Hz, 15 H, H-15), 1.73 (d, J = 11.0 Hz, 3 H, H-9), 1.92 (d, J = 11.6 Hz, 3 H, H-9), 2.32 (s, 3 H, H-6), 2.37 (s, 3 H, H-7), 2.45 (s, 3 H, H-7), 6.93 (s, 1 H, H-4), 7.04 (s, 1 H, H-4), 7.52–7.57 (m, 2 H, H-Ar), 7.57–7.62 (m, 3 H, H-Ar), 7.63–7.71 (m, 5 H, H-Ar), 7.76 (d, J = 23.0 Hz, 1 H, H-1).

^{13}C-NMR (150.90 MHz, $CDCl_3$): δ (ppm) = 9.37 (s, 5 C, C-15), 20.71 (s, 1 C, C-6), 20.89 (s, 1 C, C-7), 24.20 (d, J = 0.9 Hz, 1 C, C-9), 25.27 (s, 1 C, C-7), 25.69 (d, J = 3.9 Hz, 1 C, C-9), 52.98 (d, J = 31.1 Hz, 1 C, C-8), 96.63 (d, J = 2.4 Hz, 5 C, C-14), 121.02 (q, J = 320.8 Hz, 1 C, C-16), 124.47 (d, J = 64.7 Hz, 1 C, C-10), 128.80 (d, J = 11.4 Hz, 2 C, C-Ar), 129.10 (br s, 4 C, C-Ar), 129.47 (s, 1 C, C-3), 129.96 (s, 1 C, C-4), 130.53 (d, J = 49.5 Hz, 1 C, C-10), 131.03 (s, 1 C, C-4), 131.25 (s, 1 C, C-3), 132.63 (d, J = 2.6 Hz, 1 C, C-13), 132.90 (d, J = 2.6 Hz, 1 C, C-13), 133.69 (d, J = 9.5 Hz, 2 C, C-Ar), 138.59 (s, 1 C, C-5), 146.63 (s, 1 C, C-2), 190.74 (d, J = 10.9 Hz, 1 C, C-1).

^{31}P-NMR (242.94 MHz, $CDCl_3$): δ (ppm) = 39.20 (s, 1 P).

EA ($C_{36}H_{45}F_3NO_3PSIIr$): calcd. C: 44.26 %, H: 4.44 %, N: 1.43 %; found: C: 43.23 %, H: 5.04 %, N: 1.03 %.

HR-MS (ESI$^+$): [M−OTf]$^+$ = $C_{35}H_{45}NPIIr$ calcd.: 828.1802 found: 828.1788.

Compound [7-Rh(cod)]BF_4

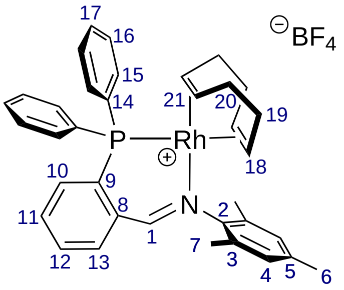

[**7**-Rh(cod)]BF_4

yield: red crystalline solid (464.4 mg, 659 µmol, 92 %, GP 3).

^{1}H-NMR (600.13 MHz, $CDCl_3$): δ (ppm) = 2.03−2.10 (m, 2 H, H-19/H-20), 2.11 (s, 6 H, H-7), 2.12−2.18 (m, 2 H, H-19/H-20), 2.31−2.40 (m, 2 H, H-19/H-20), 2.43−2.52 (m, 2 H, H-19/H-20), 2.56 (s, 3 H, H-6), 3.73−3.78 (m, 2 H, H-21), 4.48−4.54 (m, 2 H, H-18), 6.87 (s, 2 H, H-4), 7.32 (dd, J = 8.6 Hz, J = 7.7 Hz, 1 H, H-10), 7.41 (dd, J = 11.1 Hz, J = 7.8 Hz, 4 H, H-15), 7.53 (td, J = 7.6 Hz, J = 2.3 Hz, 4 H, H-16), 7.58 (td, J = 7.3 Hz, J = 1.5 Hz, 2 H, H-17), 7.68−7.72 (m, 1 H, H-11), 7.87−7.90 (m, 1 H, H-12), 7.91−7.94 (m, 1 H, H-13), 7.95 (d, J = 2.8 Hz, 1 H, H-1).

^{13}C-NMR (150.90 MHz, $CDCl_3$): δ (ppm) = 19.30 (s, 2 C, C-7), 20.94 (s, 1 C, C-6), 28.64 (d, J = 1.5 Hz, 2 C, C-19/C-20), 32.01 (d, J = 2.8 Hz, 2 C, C-19/C-20), 79.86 (d, J = 12.4 Hz, 2 C, C-21), 109.96 (dd, J = 10.4 Hz, J = 6.7 Hz, 2 C, C-18), 124.38 (d, J = 40.0 Hz, 1 C, C-13), 126.93 (d, J = 47.5 Hz, 2 C, C-9), 129.16 (s, 2 C, C-3), 129.57 (d, J = 10.5 Hz, 4 C, C-16), 129.86 (s, 2 C, C-4), 132.25 (d, J = 2.8 Hz, 2 C, C-17), 133.51 (d, J = 2.4 Hz, 1 C, C-12), 133.75 (d, J = 11.2 Hz, 4 C, C-15), 134.26 (s, 1 C, C-10), 134.85 (d, J = 6.6 Hz, 1 C, C-11), 136.41 (d, J = 17.5 Hz, 1 C, C-8), 137.44 (s, 1 C, C-5), 139.77 (d, J = 8.9 Hz, 1 C, C-13), 147.75 (s, 1 C, C-2), 171.72 (d, J = 8.1 Hz, 1 C, C-1).

^{31}P-NMR (242.94 MHz, $CDCl_3$): δ (ppm) = 30.92 (d, J = 152.91 Hz).

EA ($C_{36}H_{38}RhNPBF_4$): calcd. C: 61.30 %, H: 5.43 %, N: 1.99 %; found: C: 56.91 %, H: 5.30 %, N: 1.88 %.

HR-MS (FAB+): $[M{-}BF_4]^+$ = $C_{36}H_{38}RhNP^+$ calcd.: 618.1791 found: 618.1818.

Compound [7-Ir(cod)]OTf

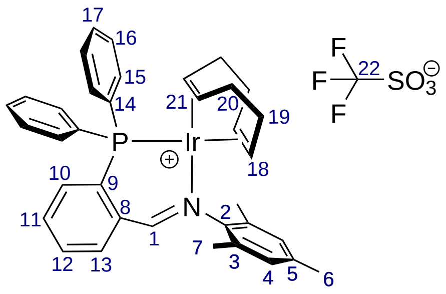

[7-Ir(cod)]OTf

yield: black crystalline solid (925.7 mg, 1.08 mmol, 85 %, GP 2).

^{1}H-NMR (600.13 MHz, $CDCl_3$): δ (ppm) = 1.87—1.98 (m, 4 H, H-19/H-20), 2.14 (s, 6 H, H-7), 2.21 (m, 2 H, H-19/H-20), 2.28 (m, 2 H, H-19/H-20), 2.30 (s, 3 H, H-6), 3.42—3.47 (m, 2 H, H-21), 4.22—4.29 (m, 2 H, H-18), 6.91 (s, 2 H, H-4), 7.38—7.44 (m, 5 H, H-15, H-10), 7.51—7.55 (m, 4 H, H-16), 7.56—7.61 (tdd, J = 7.5 Hz, J = 1.7 Hz, J = 1.2 Hz, 2 H, H-17), 7.77 (tdd, J = 7.6 Hz, J = 1.2 Hz, J = 1.2 Hz, 1 H, H-11), 7.90 (tdd, J = 7.6 Hz, J = 1.3 Hz, J = 1.3 Hz, 1 H, H-12), 8.02 (ddd, J = 7.7 Hz, J = 4.3 Hz, J = 1.2 Hz, 1 H, H-13), 8.12 (s, 1 H, H-1).

^{13}C-NMR (150.90 MHz, $CDCl_3$): δ (ppm) = 19.28 (s, 2 C, C-7), 20.90 (s, 1 C, C-6), 29.45 (d, J = 2.2 Hz, 2 C, C-19/C-20), 32.32 (d, J = 3.8 Hz, 2 C, C-19/C-20), 65.76 (s, 2 C, C-21), 98.66 (d, J = 12.0 Hz, 2 C, C-18), 120.95 (q, J = 320.8 Hz, 1 C, C-22), 125.03 (d, J = 47.7 Hz, 1 C, C-9), 126.47 (d, J = 55.6 Hz, 2 C, C-14), 129.49 (d, J = 10.8 Hz, 4 C, C-16), 129.74 (s, 2 C, C-4), 129.76 (s, 2 C, C-3), 132.49 (d, J = 2.8 Hz, 1 C, C-17), 133.68 (d, J = 2.5 Hz, 1 C, C-10), 134.04 (d, J = 11.0 Hz, 4 C, C-15), 134.41 (d, J = 2.0 Hz, 1 C, C-12), 135.46 (d, J = 7.1 Hz, 1 C, C-11), 136.69 (d, J = 5.7 Hz, 1 C, C-8), 138.07 (s, 1 C, C-2), 140.59 (d, J = 9.1 Hz, 1C, C-9), 147.25 (d, J = 5.6 Hz, 1C, C-6), 172.50 (d, J = 7.2 Hz, 1C, C-7).

^{31}P-NMR (242.94 MHz, $CDCl_3$): δ (ppm) = 16.74 (s).

EA ($C_{37}H_{38}F_3NO_3PSIr$): calcd. C: 51.86 %, H 4.47 %, N 1.63 %; found: C: 49.69 %, H: 4.53 %, N: 1.59 %.

HR-MS (FAB+): $[M{-}OTf]^+ = C_{36}H_{38}IrNP^+$ calcd.: 708.2366 found: 708.2393.

Compound [7-Pd(2-Me-allyl)]OTf

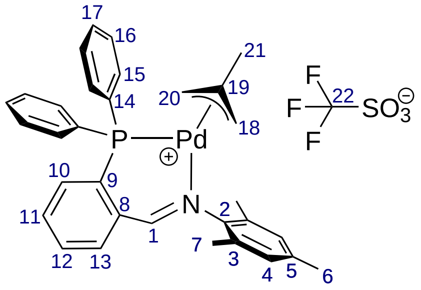

[**7**-Pd(2-Me-allyl)]OTf

yield: yellow solid (445.0 mg, 620 µmol, 49 %, GP 2).

^{1}H-NMR (600.13 MHz, $CDCl_3$): δ (ppm) = 1.86 (s, 3 H, H-7), 1.94 (s, 3 H, H-21), 2.04 (s, 3 H, H-7), 2.29 (s, 3 H, H-6), 2.82–2.89 (m, 1 H, H-20), 3.25–3.26 (m, 1 H, H-20), 3.35 (dd, J = 5.8 Hz, J = 3.1 Hz, 1 H, H-18), 3.51 (d, J = 9.6 Hz, 1 H, H-18), 6.86 (s, 1 H, H-4), 6.90 (s, 1 H, H-4), 7.21 (dd, J = 10.6 Hz, J = 7.7 Hz, 1 H, H-10), 7.23–7.28 (m, 2 H, H-15), 7.37—7.42 (m, 2 H, H-15), 7.50–7.61 (m, 6 H, H-17, H-16), 7.70 (t, J = 7.6 Hz, 1 H, H-11), 7.85 (t, J = 7.6 Hz, 1 H, H-12), 7.93 (dd, J = 6.9 Hz, J = 4.8 Hz, 1 H, H-13), 8.18 (d, J = 2.3 Hz, 1 H, H-1).

^{13}C-NMR (150.90 MHz, $CDCl_3$): δ (ppm) = 18.20 (s, 1 C, C-7), 18.42 (s, 1 C, C-7), 20.93 (s, 1 C, C-6), 24.03 (s, 1 C, C-21), 56.06 (d, J = 3.8 Hz, 1 C, C-20), 83.13 (d, J = 30.3 Hz, 1 C, C-18), 120.90 (q, J = 320.8 Hz, 1 C, C-22), 124.26 (d, J = 37.3 Hz, 1 C, C-9), 126.90 (s, 1 C, C-3),127.00 (s, 1 C, C-3), 128.36 (s, 1 C, C-14), 129.17 (s, 1 C, C-14), 129.35 (s, 1 C, C-4), 129.67 (s, 1 C, C-4), 129.88 (d, J = 11.0 Hz, 2 C, C-16), 129.95 (d, J = 11.0 Hz, 2 C, C-16), 132.26 (d, J = 2.8 Hz, 1 C, C-17), 132.38 (d, J = 2.8 Hz, 1 C, C-17), 133.27 (d, J = 14.0 Hz, 2 C, C-15), 133.41 (d, J = 2.5 Hz, 1 C, C-12), 133.57 (d, J = 13.9 Hz, 2 C, C-15), 134.94 (d, J = 6.7 Hz, 1 C, C-11), 135.45 (s, 1 C, C-10), 136.22 (d, J = 15.7 Hz, 1 C, C-8), 136.75 (s, 1 C, C-5), 138.82 (d, J = 5.7 Hz, 1 C, C-19), 139.36 (d, J = 9.0 Hz, 1 C, C-13), 152.85 (s, 1 C, C-2), 169.91 (d, J = 5.3 Hz, 1 C, C-1).

^{31}P-NMR (242.94 MHz, $CDCl_3$): δ (ppm) = 23.97 (s).

EA ($C_{33}H_{33}F_3NO_3PPd$): calcd. C: 55.20 %, H: 4.63 %, N: 1.95 %; found: C: 55.80 %, H: 5.20 %, N: 1.89 %.

HR-MS (FAB+): $[M{-}OTf]^+$ = $C_{32}H_{33}PdNP^+$ calcd.: 568.1385 found: 568.1400.

3 VT-NMR Studies

Compound [**2a**-PdCl]$_2$(BF_4)$_2$ is dimeric in the solid state, with two chlorides bridging the cationic palladium centers. Its ^{31}P-NMR spectrum in CD_2Cl_2 at room temperature features a single, broad resonance, whereas in $CDCl_3$ solution, three broad signals were found. To clarify these findings, a variable-temperature NMR study of this compound in dichloromethane was conducted, revealing that at low temperatures three species can be distinguished in solution (Figure 1).

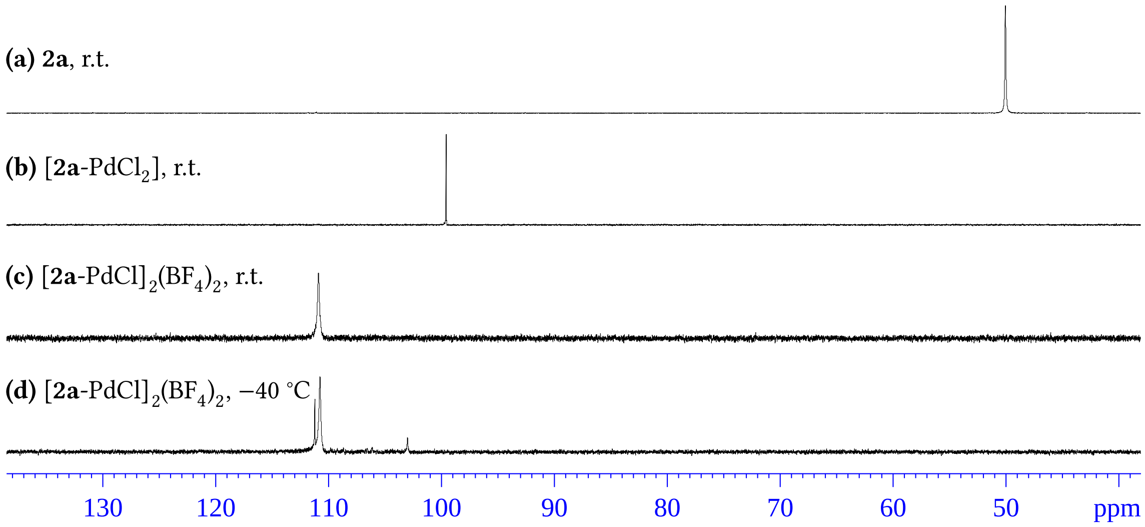

Figure 1: ^{31}P-NMR spectra of **2a**, [**2a**-PdCl$_2$] and variable-temperature ^{31}P-NMR study of the dimeric complex [2a-PdCl]$_2$(BF_4)$_2$. Solvents used: (a) THF-d_8, (b)–(d) dichloromethane-d_2.

This is in line with a solvent-dependent equilibrium between dimeric and monomeric solvated T-shaped stereoisomers, although an additional stabilizing coordination of the BF_4 anion is also possible [20].

4 X-Ray Crystal Structure Determinations

Crystal data and details of the structure determinations are compiled in Tables 1–4. Full shells of intensity data were collected at low temperature with Agilent Technologies Supernova E (Mo- or Cu-K_α radiation, microfocus X-ray tube, multilayer mirror optics) or Bruker AXS Smart 1000 (Mo-K_α radiation, sealed X-ray tube, graphite monochromator) CCD diffractometers. Data were corrected for air and detector absorption, Lorentz and polarization effects [21–23]; absorption by the crystal was treated with a semiempirical multiscan method (data collected with the Bruker instrument) [24–26] or numerically (data collected with the Agilent instrument, Gaussian grid) [21, 22, 27]. For datasets collected with the microfocus tube(s) an illumination correction was performed [28, 29]. The structures were solved by intrinsic phasing (for [**2b**-Ir(cod)]$BF_4 \cdot 0.5\,CH_2Cl_2 \cdot C_7H_8$) [30–32], by direct methods with dual-space recycling (for [**2a**-Ir(cod)]$BF_4 \cdot CH_2Cl_2$) [33, 34], by the heavy atom method combined with structure expansion by direct methods applied to difference structure factors (for [**7**-Rh(cod)]$BF_4 \cdot CH_2Cl_2$) [35, 36], or by the charge flip procedure (all other structures) [37, 38]. Refinement was carried out by full-matrix least squares methods based on F^2 against all unique reflections [39–41]. All non-hydrogen atoms were given anisotropic displacement parameters. Hydrogen atoms were generally input at calculated positions and refined with a riding model. When justified by the quality of the data, the positions of some hydrogen atoms were taken from difference Fourier synthesis and refined. When found necessary, disordered groups and/or solvent molecules were subjected to suitable geometry and adp restraints. The two independent complex cations in the structures of [**2b**-M(cod)]$BF_4 \cdot 0.5\,CH_2Cl_2 \cdot 0.5\,C_7H_8$ (M = Rh, Ir) are related by a pseudosymmetry translation. The symmetry is however broken by the toluene solvent molecule.

Due to severe disorder and/or fractional occupancy, electron density attributed to solvent of crystallization was removed from the structures of [**2a**-Cp*IrI]$BF_4 \cdot 1.5\,CH_2Cl_2$ and [**7**-Rh(cod)]$BF_4 \cdot 1.x\,CH_2Cl_2$ with the BYPASS procedure [42, 43], as implemented in PLATON (SQUEEZE) [44, 45]. Partial structure factors from the solvent masks were included in the refinement as separate contributions to F_{obs}.

CCDC 1451416–1451427 contain the supplementary crystallographic data for this paper. These data can be obtained free of charge from The Cambridge Crystallographic Data Centre *via* www.ccdc.cam.ac.uk/data_request/cif.

Table 1: Details of the crystal structure determinations of [**2a**-$PdCl_2$], [**2a**-PdCl]$_2$(BF_4)$_2$ and [**2a**-Rh(cod)]BF_4.

Compound	[**2a**-$PdCl_2$] · 3 CH_2Cl_2	[**2a**-PdCl]$_2$(BF_4)$_2$ · CH_2Cl_2 · 0.5 C_7H_8	[**2a**-Rh(cod)]BF_4 · 1.x CH_2Cl_2
Empirical formula	$C_{34}H_{39}Cl_8N_2PPd$	$C_{66.5}H_{72}B_2Cl_4F_8N_4P_2Pd_2$	$C_{40}H_{47}BCl_2F_4N_2PRh$
Formula weight M_r (g/mol)	896.64	1517.44	847.38
Crystal system	monoclinic	triclinic	triclinic
Space group	$P\ 2_1/c$ (IT Nr. 14)	$P\ \bar{1}$ (IT Nr. 2)	$P\ \bar{1}$ (IT Nr. 2)
a (Å)	11.63996(6)	11.291(6)	12.435(5)
b (Å)	22.90211(13)	13.841(7)	15.735(7)
c (Å)	14.66629(6)	21.603(10)	19.553(8)
α (°)		82.724(8)	89.234(11)
β (°)	92.3283(4)	80.839(13)	89.261(14)
γ (°)		87.563(12)	89.370(9)
V (Å^3)	3906.51(3)	3305(3)	3825(3)
Z	4	2	4
F_{000}	1816	1542	1744
d_c (Mg·m^{-3})	1.525	1.525	1.471
X-radiation, λ (Å)	Cu-K_α, 1.54184	Mo-K_α, 0.71073	Mo-K_α, 0.71073
μ (mm^{-1})	9.464	0.821	0.679
Transmission factors: max, min	0.828, 0.244	0.8828, 0.8021	0.9039, 0.8467
Data collect. temp. (K)	120(1)	100(1)	100(1)
θ range for data collection (°)	3.6 to 71.0	1.9 to 32.4	1.6 to 31.5
Index ranges h, k, l	−14 ... 14, −27 ... 25, −17 ... 17	−16 ... 16, −20 ... 20, −29 ... 31	−18 ... 18, −22 ... 23, −28 ... 28
Reflections measured	246179	65911	98486
Independent refl.[R_{int}]	7511 [0.0630]	21848 [0.0414]	25065 [0.0484]
Observed refl. [$I \geq 2\sigma(I)$]	7242	17078	19118
data / restraints / parameter	7511 / 0 / 424	21848 / 222 / 876	25065 / 168 / 983
GooF on F^2	1.058	1.024	1.021
$R\ [F > 4\sigma(F)]\ R(F),\ wR(F^2)$	0.0351, 0.0850	0.0465, 0.1082	0.0399, 0.0891
R (all data) $R(F),\ wR(F^2)$	0.0364, 0.0859	0.0683, 0.1188	0.0617, 0.0990
Diff. density: rms, max, min (e·Å^{-3})	0.080, 1.760, −1.075	0.128, 2.234, −1.702	0.104, 1.590, −1.039
Diffractometer	Agilent Supernova-E	Bruker AXS Smart 1000	Bruker AXS Smart 1000

Table 2: Details of the crystal structure determinations of [**2b**-Rh(cod)]BF_4, [**2a**-Ir(cod)]BF_4 and [**2b**-Ir(cod)]BF_4.

Compound	[**2b**-Rh(cod)]BF_4 · 0.5 CH_2Cl_2 · C_7H_8	[**2a**-Ir(cod)]BF_4 · CH_2Cl_2	[**2b**-Ir(cod)]BF_4 · 0.5 CH_2Cl_2 · C_7H_8
Empirical formula	$C_{49}H_{62}BClF_4N_2PRh$	$C_{40}H_{47}BCl_2F_4IrN_2P$	$C_{49}H_{62}BClF_4IrN_2P$
Formula weight M_r (g/mol)	935.14	936.67	1024.43
Crystal system	triclinic	triclinic	triclinic
Space group	$P\,\bar{1}$ (IT Nr. 2)	$P\,\bar{1}$ (IT Nr. 2)	$P\,\bar{1}$ (IT Nr. 2)
a (Å)	10.903(4)	12.47669(19)	10.95376(6)
b (Å)	17.768(7)	15.7562(2)	17.76136(11)
c (Å)	24.913(9)	19.5779(3)	24.90835(15)
α (°)	107.751(13)	89.2424(12)	107.7617(5)
β (°)	96.045(9)	89.4178(12)	95.9466(5)
γ (°)	96.260(13)	89.6008(12)	96.1981(5)
V (Å^3)	4520(3)	3844.17(10)	4540.08(5)
Z	4	4	4
F_{000}	1952	1872	2080
d_c (Mg·m^{-3})	1.374	1.618	1.499
X-radiation, λ (Å)	Mo-K_α, 0.71073	Mo-K_α, 0.71073	Mo-K_α, 0.71073
μ (mm^{-1})	0.525	3.705	3.087
Transmission factors: max, min	0.7464, 0.6974	0.918, 0.569	0.825, 0.591
Data collect. temp. (K)	100(1)	120(1)	120(1)
θ range for data collection (°)	0.9 to 32.5	3.3 to 26.4	3.2 to 32.9
Index ranges h, k, l	−15 ... 15, −26 ... 26, −37 ... 37	−15 ... 15, −19 ... 19, −24 ... 24	−16 ... 16, −26 ... 26, −37 ... 38
Reflections measured	117410	80938	658461
Independent refl. [R_{int}]	30412 [0.0376]	15685 [0.0611]	32099 [0.0740]
Observed refl. [$I \geq 2\sigma(I)$]	22684	13294	29136
data / restraints / parameter	30412 / 100 / 1096	15685 / 302 / 959	32099 / 100 / 1096
GooF on F^2	1.029	1.196	1.225
R [$F > 4\sigma(F)$] $R(F)$, $wR(F^2)$	0.0427, 0.1030	0.0511, 0.1092	0.0460, 0.0716
R (all data) $R(F)$, $wR(F^2)$	0.0634, 0.1155	0.0620, 0.1132	0.0549, 0.0739
Diff. density: rms, max, min (e·Å^{-3})	0.106, 2.207, −1.111	0.158, 2.317, −2.827	0.116, 1.631, −1.997
Diffractometer	Bruker AXS Smart 1000	Agilent SuperNova-E	Agilent SuperNova-E

TABLE 3: Details of the crystal structure determinations of [**2a**-Cp*IrI]BF_4, [**5**-Pd(2-Me-allyl)]OTf and [**5**-Rh(cod)]BF_4.

Compound	[**2a**-Cp*IrI]BF_4 · 1.5 CH_2Cl_2	[**5**-Pd(2-Me-allyl)]OTf	[**5**-Rh(cod)]BF_4
Empirical formula	$C_{42.5}H_{52}BCl_3F_4IIrN_2P$	$C_{30}H_{35}F_3NO_3PPdS$	$C_{33}H_{40}BF_4NPRh$
Formula weight M_r (g/mol)	1134.09	684.02	671.35
Crystal system	triclinic	orthorhombic	monoclinic
Space group	$P\ \bar{1}$ (IT Nr. 2)	$P\ b\ c\ a$	$P\ 2_1/c$ (IT Nr. 14)
a (Å)	11.539(4)	17.413(9)	12.941(5)
b (Å)	11.740(4)	17.673(8)	12.948(6)
c (Å)	16.334(6)	19.974(10)	19.111(8)
α (°)	101.867(10)		
β (°)	93.026(7)		106.643(10)
γ (°)	94.659(6)		
V (Å^3)	2152.9(14)	6147(5)	3068(2)
Z	2	8	4
F_{000}	1097	2800	1384
d_c (Mg·m^{-3})	1.749	1.478	1.453
X-radiation, λ (Å)	Mo-K_α, 0.71073	Mo-K_α, 0.71073	Mo-K_α, 0.71073
μ (mm^{-1})	4.092	0.773	0.657
Transmission factors: max, min	0.3391, 0.2665	0.7464, 0.6717	0.8623, 0.8050
Data collect. temp. (K)	100(1)	100(1)	100(1)
θ range for data collection (°)	2.0 to 32.5	1.9 to 26.4	1.9 to 32.5
Index ranges h, k, l	−17 ... 17, −17 ... 17, −24 ... 24	−21 ... 21, −22 ... 22, −24 ... 24	−19 ... 19, −19 ... 19, −28 ... 28
Reflections measured	55403	107373	78525
Independent refl. [R_{int}]	14467 [0.0243]	6277 [0.0321]	10718 [0.0300]
Observed refl. [$I \geq 2\sigma(I)$]	13900	5102	9691
data / restraints / parameter	14467 / 0 / 498	6277 / 12 / 382	10718 / 21 / 390
GooF on F^2	1.052	1.196	1.047
R [$F > 4\sigma(F)$] $R(F)$, $wR(F^2)$	0.0186, 0.0463	0.0393, 0.0765	0.0245, 0.0596
R (all data) $R(F)$, $wR(F^2)$	0.0200, 0.0470	0.0569, 0.0929	0.0288, 0.0629
Diff. density: rms, max, min (e·Å^{-3})	0.096, 1.340, −1.700	0.109, 1.702, −0.553	0.073, 1.024, −0.754
Diffractometer	Bruker AXS Smart 1000	Bruker AXS Smart 1000	Bruker AXS Smart 1000

TABLE 4: Details of the crystal structure determinations of [**5**-Ir(cod)]OTf, [**5**-Cp*IrI]OTf and [**7**-Rh(cod)]BF_4.

Compound	[**5**-Ir(cod)]OTf	[**5**-Cp*IrI]OTf · $CHCl_3$	[**7**-Rh(cod)]BF_4 · CH_2Cl_2
Empirical formula	$C_{34}H_{40}F_3IrNO_3PS$	$C_{37}H_{44}Cl_3F_3IIrNO_3PS$	$C_{37}H_{40}BCl_2F_4NPRh$
Formula weight M_r (g/mol)	822.90	1096.21	790.29
Crystal system	monoclinic	monoclinic	triclinic
Space group	$P\ 2_1/n$ (IT Nr. 14)	$P\ 2_1/n$ (IT Nr. 14)	$P\ 1$ (IT Nr. 1)
a (Å)	13.001(6)	17.335(7)	9.78588(12)
b (Å)	18.967(8)	9.113(4)	14.13707(16)
c (Å)	13.378(5)	25.210(11)	20.6977(3)
α (°)			105.6171(11)
β (°)	94.921(9)	101.562(9)	96.4680(11)
γ (°)			104.3606(10)
V ($Å^3$)	3287(2)	3902(3)	2621.65(6)
Z	4	4	3
F_{000}	1640	2144	1212
d_c ($Mg·m^{-3}$)	1.663	1.866	1.502
X-radiation, λ (Å)	Mo-K_α, 0.71073	Mo-K_α, 0.71073	Mo-K_α, 0.71073
μ (mm^{-1})	4.227	4.565	0.737
Transmission factors: max, min	0.4949, 0.3820	0.7464, 0.5181	0.977, 0.926
Data collect. temp. (K)	100(1)	100(1)	120(1)
θ range for data collection (°)	2.1 to 32.5	2.4 to 32.5	3.2 to 32.9
Index ranges h, k, l	−19 ... 18, −28 ... 28, −19 ... 19	−25 ... 25, −13 ... 13, −38 ... 37	−14 ... 14, −21 ... 21, −30 ... 30
Reflections measured	83623	97716	87324
Independent refl. [R_{int}]	11337 [0.0345]	13477 [0.0438]	34788 [0.0516]
Observed refl. [$I \geq 2\sigma(I)$]	10320	11946	30637
data / restraints / parameter	11337 / 0 / 417	13477 / 0 / 473	34788 / 169 / 1251
GooF on F^2	1.047	1.026	1.026
R [$F > 4\sigma(F)$] $R(F)$, $wR(F^2)$	0.0181, 0.0397	0.0252, 0.0590	0.0446, 0.0896
R (all data) $R(F)$, $wR(F^2)$	0.0223, 0.0412	0.0321, 0.0620	0.0542, 0.0955
Diff. density: rms, max, min ($e·Å^{-3}$)	0.099, 1.596, −0.892	0.144, 1.757, −2.082	0.099, 0.905, −0.646
Absolute structure parameter			−0.005(9)
Diffractometer	Bruker AXS Smart 1000	Bruker AXS Smart 1000	Agilent SuperNova-E

References

1. Armarego, W. L. F.; Chai, C. L. L., *Purification of Laboratory Chemicals*, 6th ed.; Amsterdam; Boston: Elsevier/Butterworth-Heinemann: 2009.
2. Gottlieb, H. E.; Kotlyar, V.; Nudelman, A. *J. Org. Chem.* **1997**, *62*, 7512–7515.
3. Fulmer, G. R.; Miller, A. J. M.; Sherden, N. H.; Gottlieb, H. E.; Nudelman, A.; Stoltz, B. M.; Bercaw, J. E.; Goldberg, K. I. *Organometallics* **2010**, *29*, 2176–2179.
4. Kuhn, K. M.; Grubbs, R. H. *Org. Lett.* **2008**, *10*, 2075–2077.
5. Krahulic, K. E.; Enright, G. D.; Parvez, M.; Roesler, R. *J. Am. Chem. Soc.* **2005**, *127*, 4142–4143.
6. Roth, T.; Vasilenko, V.; Wadepohl, H.; Wright, D. S.; Gade, L. H. *Inorg. Chem.* **2015**, *54*, 7636–7644.
7. Krackl, S.; Inoue, S.; Driess, M.; Enthaler, S. *Eur. J. Inorg. Chem.* **2011**, *2011*, 2103–2111.
8. Abdou, H. E.; Mohamed, A. A.; López-de-Luzuriaga, J. M.; Monge, M.; Fackler, J. P. *Inorg. Chem.* **2012**, *51*, 2010–2015.
9. Cotton, F. A.; Murillo, C. A.; Pascual, I. *Inorg. Chem.* **1999**, *38*, 2182–2187.
10. Capon, B.; Wu, Z. P. *J. Org. Chem.* **1990**, *55*, 2317–2324.
11. Daugulis, O.; Brookhart, M. *Organometallics* **2002**, *21*, 5926–5934.
12. Jarvis, A. G.; Sehnal, P. E.; Bajwa, S. E.; Whitwood, A. C.; Zhang, X.; Cheung, M. S.; Lin, Z.; Fairlamb, I. J. S. *Chem. Eur. J.* **2013**, *19*, 6034–6043.
13. Hoots, J. E.; Rauchfuss, T. B.; Wrobleski, D. A.; Knachel, H. C. In *Inorganic Syntheses*; John Wiley & Sons, Inc.: 2007, pp 175–179.
14. Ahlmann, M.; Walter, O. *J. Organomet. Chem.* **2004**, *689*, 3117–3131.
15. Cramer, N.; Laschat, S.; Baro, A. *Organometallics* **2006**, *25*, 2284–2291.
16. Yoshida, H.; Shirakawa, E.; Kurahashi, T.; Nakao, Y.; Hiyama, T. *Organometallics* **2000**, *19*, 5671–5678.
17. Shirakawa, E.; Nakao, Y.; Murota, Y.; Hiyama, T. *J. Organomet. Chem.* **2003**, *670*, 132–136.
18. Xue, Z.; Linh, N. T. B.; Noh, S. K.; Lyoo, W. S. *Angew. Chem. Int. Ed.* **2008**, *47*, 6426–6429.
19. Two resonances with approximate ratios 3 : 1 were detected in the ^{31}P-NMR spectrum. The side product is structurally closely related to the desired complex and a separation could not be achieved in the scope of this work. Signals of the major component are listed tentatively.
20. A similar behaviour was observed by Shaffer and Schmidt for structurally related (3-iminophosphine)palladium(II) complexes containing triflate counterions: Shaffer, A. R.; Schmidt, J. A. R. *Organometallics* **2009**, *28*, 2494–2504.
21. *CryAlisPro*, Agilent Technologies UK Ltd., Oxford, UK **2011–2014**.

22. Rigaku Oxford Diffraction, Rigaku Polska Sp.z o.o., Wrocław, Poland **2015**.
23. *SAINT*, Bruker AXS GmbH, Karlsruhe, Germany **1997–2013**.
24. Sheldrick, G. M. *SADABS*, Bruker AXS GmbH, Karlsruhe, Germany **2004–2014**.
25. Krause, L.; Herbst-Irmer, R.; Sheldrick, G. M.; Stalke D. *J. Appl. Cryst.* **2015**, *48*, 3.
26. Blessing, R. H. *Acta Cryst.* **1995**, *A51*, 33.
27. Busing, W. R.; Levy H. A. *Acta Cryst.* **1957**, *10*, 180.
28. *SCALE3 ABSPACK, CryAlisPro*, Agilent Technologies UK Ltd., Oxford, UK **2011–2014**.
29. Rigaku Oxford Diffraction, Rigaku Polska Sp.z o.o., Wrocław, Poland **2015**.
30. Sheldrick, G. M. SHELXT, University of Göttingen and Bruker AXS GmbH, Karlsruhe, Germany, **2012–2014**.
31. Ruf, M.; Noll, B. C. *Application Note SC-XRD 503*, Bruker AXS GmbH Karlsruhe, Germany, **2014**.
32. Sheldrick, G. M. *Acta Cryst.* **2015**, *A71*, 3.
33. Burla, M. C.; Caliandro, R.; Camalli, M.; Carrozzini, B.; Cascarano, G. L.; Giacovazzo, C.; Mallamo, M.; Mazzone, A.; Polidori, G.; Spagna, R. *SIR2011*, CNR IC, Bari, Italy, **2011**.
34. Burla, M. C.; Caliandro, R.; Camalli, M.; Carrozzini, B.; Cascarano, G. L.; Giacovazzo, C.; Mallamo, M.; Mazzone, A.; Polidori, G.; Spagna, R. *J. Appl. Cryst.* **2012**, *45*, 357.
35. Beurskens, P. T.; Beurskens G.; de Gelder, R.; Smits, J. M. M.; Garcia-Granda, S.; Gould, R. O. *DIRDIF-2008*, Radbound University Nijmegen, The Netherlands **2008**.
36. Beurskens, P. T. in: Sheldrick, G. M.; Krüger, C.; Goddard, R. (eds.) *Crystallographic Computing 3*, Clarendon Press, Oxford, UK, **1985**, p. 216.
37. Palatinus, L. *SUPERFLIP*, EPF Lausanne, Switzerland and Fyzikální ústav AV ČR, v. v. i., Prague, Czech Republic, **2007–2014**.
38. Palatinus, L.; Chapuis, G. *J. Appl. Cryst.* **2007**, *40*, 786.
39. Sheldrick, G. M. *SHELXL-20xx*, University of Göttingen and Bruker AXS GmbH, Karlsruhe, Germany **2012–2014**.
40. Sheldrick, G. M. *Acta Cryst.* **2008**, *A64*, 112.
41. Sheldrick, G. M. *Acta Cryst.* **2015** *C71*, 3.
42. v. d. Sluis, P.; Spek, A. L. *Acta Cryst.* **1990**, *A46*, 194.
43. Spek, A. L. *Acta Cryst.* **2015**, *C71*, 9.
44. Spek, A. L. *PLATON*, Utrecht Unversity, The Netherlands; http://www.platonsoft.nl.
45. Spek, A. L. *J. Appl. Cryst.* **2003**, *36*, 7.
